# Characteristics of Prescription Opioid Analgesics in Pregnancy and Risk of Neonatal Opioid Withdrawal Syndrome in Newborns

**DOI:** 10.1001/jamanetworkopen.2022.28588

**Published:** 2022-08-24

**Authors:** Daina B. Esposito, Krista F. Huybrechts, Martha M. Werler, Loreen Straub, Sonia Hernández-Díaz, Helen Mogun, Brian T. Bateman

**Affiliations:** 1 Department of Epidemiology, Boston University School of Public Health, Boston, Massachusetts; 2 Division of Pharmacoepidemiology and Pharmacoeconomics, Brigham and Women’s Hospital, Boston, Massachusetts; 3 Department of Epidemiology, Harvard T.H. Chan School of Public Health, Boston, Massachusetts; 4 Department of Anesthesia, Perioperative and Pain Medicine, Stanford University School of Medicine, Stanford, California

## Abstract

**Importance:**

Prescription opioids are often used during pregnancy even though they are associated with neonatal opioid withdrawal syndrome (NOWS). Most studies of adverse outcomes of opioid use for pain have assessed only the class-wide outcome despite the pharmacodynamic and pharmacokinetic heterogeneity across opioid medications.

**Objective:**

To compare the risk of NOWS across common types of opioids when prescribed as monotherapy during the last 3 months of pregnancy.

**Design, Setting, and Participants:**

This cohort study analyzed administrative claims data of Medicaid-insured mothers and newborns in 46 states and Washington DC from January 1, 2000, through December 31, 2014. Participants were mothers with 2 or more dispensed opioid prescriptions within 90 days before delivery and their eligible live-born neonates. Data were analyzed from February 2020 to March 2021.

**Exposure:**

Different types of opioid medications were compared by agonist strength (strong vs weak) and half-life (medium vs short and long vs short) of the opioid active ingredient.

**Main Outcomes and Measures:**

The primary outcome was NOWS, which was identified using an *International Classification of Diseases, Ninth Revision, Clinical Modification* diagnostic code in the 30 days after delivery. Relative risks (RRs) were adjusted for an exposure propensity score, including demographic characteristics, comorbidities, other medication use, and opioid treatment characteristics (including morphine milligram equivalents), using fine stratification.

**Results:**

The cohort comprised 48 202 opioid-exposed pregnancies with live newborns. A total of 1069 neonates (2.2%) had NOWS and 559 (1.2%) had severe NOWS. Opioid exposure during pregnancy included 16 202 pregnancies exposed to codeine, 4540 to oxycodone, 1244 to tramadol, 260 to methadone (dispensed for pain), 90 to hydromorphone, and 63 to morphine compared with 25 710 exposed to hydrocodone. Demographic characteristics varied across opioids, with tramadol, oxycodone, methadone, hydromorphone, and morphine being more commonly dispensed at older maternal age (≥35 years). Compared with hydrocodone, codeine had a lower adjusted RR of NOWS (0.57; 95% CI, 0.46-0.70), with a similar adjusted RR for tramadol (RR, 1.06; 95% CI, 0.73-1.56), and 2- to 3-fold higher adjusted RRs for oxycodone (1.87; 95% CI, 1.66-2.11), morphine (2.84; 95% CI, 1.30-6.22), methadone (3.02; 95% CI, 2.45-3.73), and hydromorphone (2.03; 95% CI, 1.09-3.78). Strong agonists were associated with a higher risk of NOWS than weak agonists (RR, 1.97; 95% CI, 1.78-2.17), and long half-life opioids were associated with an increased risk compared with short half-life products (RR, 1.33; 95% CI, 1.12-1.56). Findings were consistent across sensitivity and subgroup analyses.

**Conclusions and Relevance:**

Results of this study show higher risk of NOWS and severe NOWS among neonates with in utero exposure to strong agonists and long half-life prescription opioids. Information on the opioid-specific risk of NOWS may help prescribers select opioids for pain management in late stages of pregnancy.

## Key Points

**Question:**

Does risk of neonatal opioid withdrawal syndrome (NOWS) after in utero exposure to prescription opioids vary across commonly prescribed types of opioids?

**Findings:**

In this cohort study of 48 202 opioid-exposed pregnancies with live-born neonates, strong agonists were associated with a higher risk of NOWS compared with weak agonists, and long half-life opioids were associated with an increased risk compared with short half-life products. These associations were independent of morphine milligram equivalents.

**Meaning:**

The study suggests that knowing the varying opioid-specific risk of NOWS associated with in utero exposure may help prescribers select opioids for pain management in late stages of pregnancy.

## Teaser

This cohort study evaluates the risk of opioid withdrawal syndrome in newborns according to the agonist strength, half-life, and active ingredients in the prescription opioids dispensed to their mothers during pregnancy.

## Introduction

In the US, opioids are dispensed during pregnancy to approximately 20% of Medicaid beneficiaries and 14% of commercial insurance beneficiaries.^1,2^ However, opioids have been shown to cross the placenta and increase health risks to both the mother and unborn child.^3,4,5^

A key concern is neonatal opioid withdrawal syndrome (NOWS), a complex and variable syndrome that can range from irritability and mild tremor to seizures, fever, and excessive weight loss. Newborns with NOWS are at a higher risk for prolonged hospitalization and intensive care unit admission, birth complications, and disrupted bonding.^6,7^ Along with the growth of opioid exposure in pregnancy,^1^ the prevalence of NOWS has increased dramatically in the US,^8^ from 1.2 to 8.8 per 1000 hospital births from 2000 to 2016.^9^ Previous studies have shown that risk varies substantially across factors, such as misuse of opioids, opioid dependence, nonopioid psychotropic drug use, and smoking,^10^ and that concomitant exposure to prescription opioids and psychotropic medications is associated with increased risk and severity of neonatal drug withdrawal.^11^ Exposure within 90 days before delivery has been associated with development of NOWS; however, duration and intensity of exposure throughout pregnancy could play a role.^10,11^

Despite the pharmacodynamic and pharmacokinetic differences and the potential variations in adverse effects across individual opioids,^12,13^ most studies of the association between opioids and perinatal adverse outcomes assessed opioids as a class. In this study, we aimed to compare the risk of NOWS across common types of opioids (as defined by agonist strength, half-life, and active ingredient) when prescribed as monotherapy during the last 3 months of pregnancy.

## Methods

### Setting and Population

This cohort study used the Medicaid Analytic eXtract (MAX; Centers for Medicare & Medicaid), which contains administrative billing data for Medicaid enrollees in 46 states and Washington DC from January 1, 2000, through December 31, 2014 (the most recent year for which nationwide data were available at the time of this study). The study was approved by the Brigham and Women’s Hospital Institutional Review Board, which waived the informed consent requirement because the MAX data obtained and analyzed were deidentified. We followed the Strengthening the Reporting of Observational Studies in Epidemiology (STROBE) reporting guideline.

In the MAX, pregnancies that were identified via delivery codes were linked to live-born neonates using a family case number. Medication use and medical histories were ascertained from medical encounter codes captured from health care settings and prescription dispensing from outpatient pharmacy encounters. The utility of MAX for assessing drug exposures in pregnancy has been previously demonstrated,^14,15^ and numerous analyses have been conducted in the linked mother-neonate cohort.^10,16,17,18,19,20^

Within the linked mother-neonate data set, we defined a cohort of mothers with 2 or more dispensed opioid prescriptions during the 90 days before delivery, enrollment in Medicaid starting at least 270 days before delivery and continuing for at least 30 days after delivery, and no evidence of supplemental insurance or restricted benefits. Linked neonates were required to have 30 days or more of eligibility after birth (unless they died sooner). To focus on the outcome of prescription opioid use, we excluded mothers with 1 or more pharmacy-dispensed naltrexone, naloxone, or buprenorphine; a charge code for methadone used as opioid maintenance therapy for dependence (rather than for pain)^21^; or a diagnosis of opioid use disorder or opioid overdose (possible indicator of illicit use) during the 270 days before delivery.

### Exposure

To facilitate comparisons across different opioid medications, we restricted the analysis to mothers who received only 1 type of dispensed opioid medication. At least 2 dispensed opioid prescriptions were required to decrease the risk of exposure misclassification, wherein a mother could have received an opioid that was not ultimately used. Because cumulative exposure was expected to be important when comparing opioids by active ingredient, we also excluded patients with inadequate information on dose (ie, residence in a state that did not accurately capture dose, apparent cumulative exposure less than 1 or greater than 30 000 morphine milligram equivalents [MMEs] over the 90 days before delivery, or use of formulations without equianalgesic dose information). Opioid products received by fewer than 50 qualifying pregnant individuals during the 90 days before delivery were excluded.

**Figure 1.**
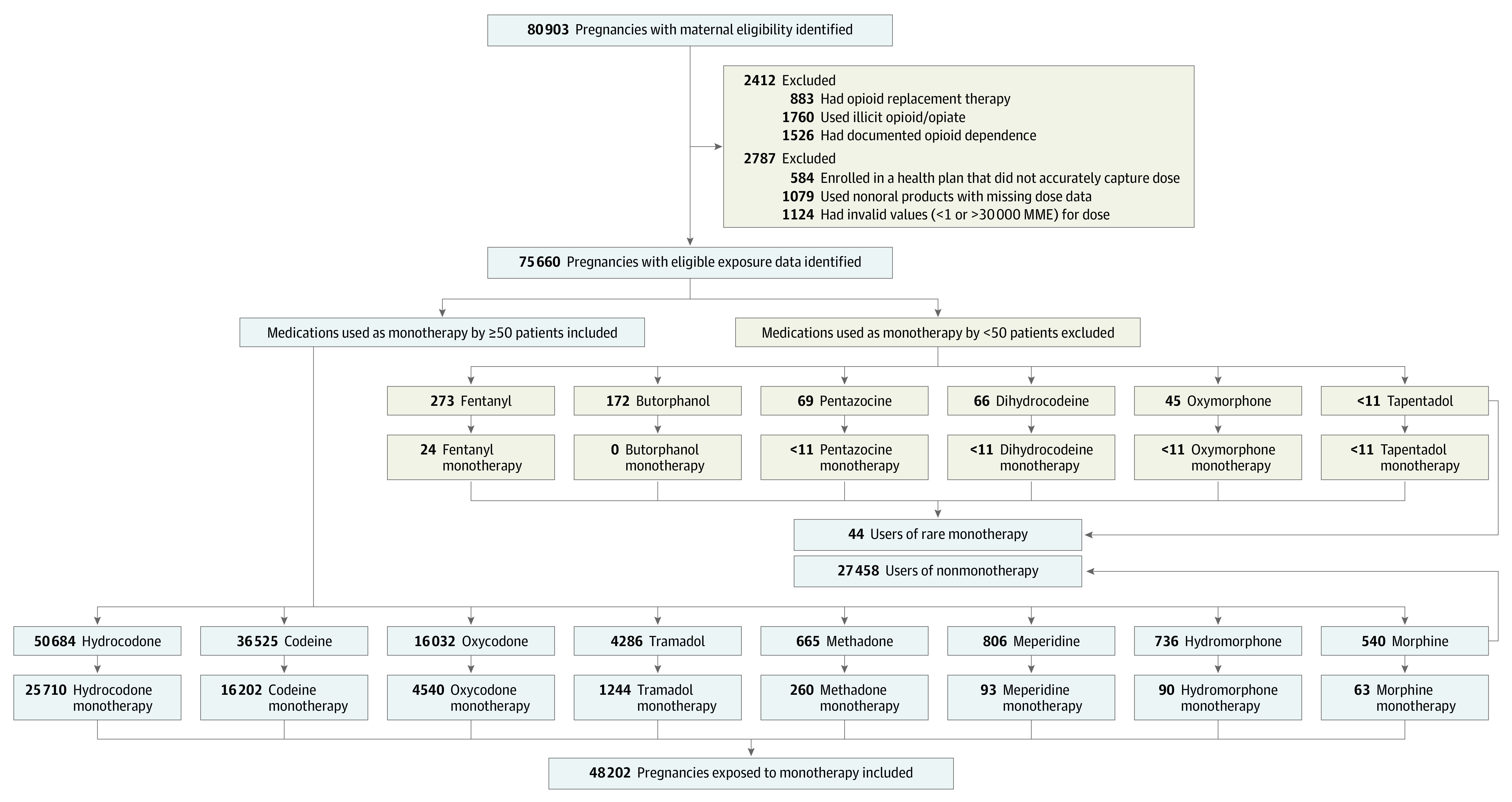
Selection of the Study Cohort MME indicates morphine milligram equivalent.

Comparisons were made by opioid agonist strength (strong vs weak) and half-life (medium vs short and long vs short) of the active ingredient, with medications categorized according to their US package inserts (Figure 1). Hydrocodone, the opioid most commonly used as monotherapy in this study population, served as the reference group for comparisons of opioids by active ingredient. Opioid treatment characteristics, including cumulative exposure in MMEs (calculated by strength, quantity dispensed, and a conversion factor), number of dispensed prescriptions received, days of supply, and exposure timing, were all assessed during the 90 days before delivery.

### Outcomes

The primary outcome was NOWS, which was defined as the presence of *International Classification of Diseases, Ninth Revision, Clinical Modification *(*ICD-9-CM*) diagnosis code 779.5x (drug withdrawal syndrome in newborn) within 30 days of delivery on administrative claims for either the mother or newborn. Although *ICD-9-CM* diagnosis code 779.5x is not specific to NOWS, known opioid exposure shortly before delivery is the most plausible exposure. Maternal claims were included when assessing NOWS given that delays in processing neonate eligibility for Medicaid can result in neonate-specific diagnosis codes being added to maternal claims. A validation study in the Mass General Brigham Healthcare System identified a positive predictive value of 91% (95% CI, 82%-97%) for NOWS overall and 100% (95% CI, 65%-100%) among neonates with intrauterine exposure to any prescribed opioid.^11^ As a proxy of severe NOWS, a secondary outcome required that the diagnosis was accompanied by an intensive care unit stay or *ICD-9-CM* diagnosis codes indicating feeding difficulties, respiratory symptoms, or seizure.^10^

### Covariates

Demographic characteristics, including race and ethnicity, were identified based on Medicaid enrollment files and defined on the date of delivery. Race and ethnicity were assessed as potential confounding factors; racial and ethnic categories included Black, Hispanic or Latinx, White, and other. Other potential confounders were defined using *ICD-9-CM* codes, *Current Procedural Terminology* codes, Healthcare Common Procedure Coding System codes, and National Drug Code numbers. The maternal comorbidity index score,^22^ psychiatric conditions (eg, depression or anxiety), conditions that may increase risk of pregnancy complications (eg, anemia, diabetes, or hypertension), and previous medication use (eg, antinausea, antibiotic, or anticonvulsant medications) were defined using data from 270 days through 90 days before delivery. Exposure to medications potentially associated with NOWS was assessed during the 90 days before delivery.

Only confounders with an absolute standardized difference of 10% or greater for at least 1 exposure contrast and the comparison of individuals with vs without NOWS among those with hydrocodone exposure were included in an exposure propensity score used for adjustment. Selected covariates are listed in the Table, and all of the covariates examined are provided in eTables 1 and 2 in the Supplement.

**Table.** Patient Characteristics by Use of Common Products as Opioid Monotherapy Medication

	Hydrocodone, No. (%)	Oxycodone			Codeine			Tramadol		
		No. (%)	Standardized difference^a^		No. (%)	Standardized difference^a^		No. (%)	Standardized difference^a^	
			Crude	Adjusted		Crude	Adjusted		Crude	Adjusted
All patients	25 710 (100)	4540 (100)	NA	NA	16 202 (100)	NA	NA	1244 (100)	NA	NA
Region of residence										
West	6024 (23.4)	579 (12.8)	−39.6	−11.9	2781 (17.2)	−22.1	−0.7	196 (15.8)	−27.5	0.2
Central	8384 (32.6)	1366 (30.1)	−7.7	1.3	7107 (43.9)	33.0	−0.6	443 (35.6)	9.0	0.9
South	9895 (38.5)	1433 (31.6)	−20.6	6.8	4686 (28.9)	−28.8	2.1	458 (36.8)	−4.9	−0.8
Northeast	1407 (5.5)	1162 (25.6)	81.8	1.2	1628 (10.1)	24.3	−1.2	147 (11.8)	32.2	−0.4
Maternal age at delivery, y										
<18	732 (2.9)	76 (1.7)	−11.2	1.3	617 (3.8)	7.6	−0.4	20 (1.6)	−11.9	−0.1
18-24	11 103 (43.2)	1574 (34.7)	−24.8	2.1	7733 (47.7)	12.9	1.6	400 (32.2)	−32.4	−0.2
25-34	12 046 (46.9)	2420 (53.3)	18.3	−4.2	6890 (42.5)	−12.3	−1.4	678 (54.5)	21.7	−0.7
≥35	1829 (7.1)	470 (10.4)	16.3	3.0	962 (5.9)	−6.7	−0.2	146 (11.7)	22.5	1.4
Year of delivery										
2000-2004	4254 (16.6)	602 (13.3)	−13.1	2.6	5495 (33.9)	57.7	−0.7	142 (11.4)	−21.0	0
2005-2009	11 120 (43.3)	1671 (36.8)	−18.6	2.3	6545 (40.4)	−8.2	−0.4	442 (35.5)	−22.4	−0.6
2010-2014	10 336 (40.2)	2267 (49.9)	27.8	−3.9	4162 (25.7)	−44.2	1.2	660 (53.1)	36.7	0.5
Race and ethnicity^b^										
Black	3777 (14.7)	888 (19.6)	18.3	−9.8	4210 (26.0)	40.1	−0.5	158 (12.7)	−8.2	−0.6
Hispanic or Latinx	1448 (5.6)	185 (4.1)	−10.3	1.0	1222 (7.5)	10.9	−0.3	74 (6.0)	1.9	0.6
White	18 725 (72.8)	3039 (66.9)	−18.2	8.6	9581 (59.1)	−41.3	0.5	913 (73.4)	1.8	−0.7
Other^c^	1760 (6.9)	428 (9.4)	13.4	−1.0	1189 (7.3)	2.7	0.1	99 (8.0)	6.0	1.4
Neonate sex										
Male	12 226 (47.6)	2143 (47.2)	−1.0	−0.7	7708 (47.6)	0.1	0.7	605 (48.6)	3.1	−0.1
Male-female twins	567 (2.2)	105 (2.3)	1.0	1.0	431 (2.7)	4.1	0.6	20 (1.6)	−6.2	−0.3
Female	12 850 (50.0)	2289 (50.4)	1.2	0.4	8010 (49.4)	−1.5	−1.0	616 (49.5)	−1.3	0.2
Other or unknown^c^	67 (0.3)	<11	−6.6	0.3	53 (0.3)	1.8	0.5	<11	−0.6	−0.1
Multiparity	19 707 (76.7)	3547 (78.1)	5.0	−4.3	12 102 (74.7)	−6.5	−0.9	999 (80.3)	12.6	−0.2
Tobacco use	2585 (10.1)	543 (12.0)	8.6	1.0	1021 (6.3)	−19.4	0.5	145 (11.7)	7.3	1.0
Maternal comorbidity index score										
0	10 920 (42.5)	1455 (32.1)	−30.7	3.2	7707 (47.6)	14.5	0.2	488 (39.2)	−9.3	0
1	6591 (25.6)	1070 (23.6)	−6.8	−4.5	4104 (25.3)	−1.0	−0.3	319 (25.6)	0	0.1
2	3999 (15.6)	851 (18.7	12.0	1.7	2230 (13.8)	−7.2	0.7	203 (16.3)	3.0	0.2
≥3	4200 (16.3)	1164 (25.6)	32.5	−0.4	2161 (13.3)	−12.0	−0.6	234 (18.8)	9.2	−0.2
Potential opioid indications										
Arthritis or arthropathies	5423 (21.1)	1116 (24.6)	11.8	2.7	2363 (14.6)	−24.1	0.4	320 (25.7)	15.5	0.1
Back and neck pain	8444 (32.8)	1910 (42.1)	27.1	3.1	3277 (20.2)	−40.8	−1.0	498 (40.0)	21.2	−0.6
Dental pain	2460 (9.6)	360 (7.9)	−8.2	1.5	1237 (7.6)	−9.8	1.2	151 (12.1)	11.7	0
Joint pain	2543 (9.9)	507 (11.2)	5.9	1.4	1042 (6.4)	−17.9	−0.3	174 (14.0)	17.9	0.7
Orthopedic injury	3555 (13.8)	655 (14.4)	2.4	0.5	1678 (10.4	−15.1	−0.1	176 (14.2)	1.3	0.8
Neuropathy or neuralgia	1699 (6.6)	520 (11.5)	24.0	2.5	511 (3.2)	−22.8	−0.3	100 (8.0)	7.8	0.5
Other chronic pain	1016 (4.0)	392 (8.6)	27.4	2.1	394 (2.4)	−12.2	0.2	88 (7.1)	19.4	−1.1
Other acute pain	3410 (13.3)	942 (20.7)	28.3	−1.3	1511 (9.3)	−17.6	−0.3	142 (11.4)	−8.0	0.4
Psychiatric conditions										
Anxiety	2591 (10.1)	539 (11.9)	8.1	−1.4	939 (5.8)	−22.5	0	154 (12.4)	10.3	0.2
Bipolar disorder	968 (3.8)	254 (5.6)	12.2	0.9	474 (2.9)	−6.6	0	60 (4.8)	7.3	0.1
Depression	2889 (11.2)	646 (14.2)	12.7	1.3	1539 (9.5)	−8.1	−1.0	162 (13.0)	7.7	0.3
Sleep disorders	495 (1.9)	121 (2.7)	7.0	2.0	173 (1.1)	−10.0	−1.2	37 (3.0)	9.5	0.5
Substance use	645 (2.5)	155 (3.4)	7.6	1.0	383 (2.4)	−1.3	−1.0	48 (3.9)	10.9	0.2
Hypertension	1240 (4.8)	304 (6.7)	11.4	2.1	598 (3.7)	−7.9	0.7	84 (6.8)	11.7	0.7
Medication use										
Medications during the 90 d before delivery										
Barbiturates	808 (3.1)	166 (3.7)	4.1	0.5	554 (3.4)	2.2	−4.8	64 (5.1)	14.2	−0.4
Benzodiazepines										
Long acting	290 (1.1)	77 (1.7)	6.8	−12.7	55 (0.3)	−13.1	−0.8	14 (1.1)	0	0.6
Short acting	1634 (6.4)	428 (9.4)	16.1	−2.6	324 (2.0)	−31.0	−0.1	111 (8.9)	13.6	−0.8
SNRI	234 (0.9)	53 (1.2)	3.6	−0.8	81 (0.5)	−6.9	−1.6	33 (2.7)	18.7	−1.1
SSRI	2551 (9.9)	477 (10.5)	2.8	−1.4	1297 (8.0)	−9.5	0	147 (11.8)	8.6	0.7
Tricyclic antidepressants	231 (0.9)	70 (1.5)	8.3	0.1	76 (0.5)	−7.4	−0.6	28 (2.3)	15.4	−0.1
Other antidepressants	602 (2.3)	146 (3.2)	7.6	0.6	289 (1.8)	−5.6	−0.9	37 (3.0)	5.5	−0.4
Other hypnotics	3349 (13.0)	735 (16.2)	12.7	0.4	1736 (10.7)	−10.2	−0.6	171 (13.8)	3.0	1.4
Medications during the baseline period										
Antinausea	11 162 (43.4)	1897 (41.8)	−4.7	4.1	5426 (33.5)	−29.0	0.6	492 (39.6)	−11.1	1.3
Anticonvulsants	1692 (6.6)	495 (10.9)	21.7	1.9	583 (3.6)	−19.2	−0.9	147 (11.8)	25.8	−1.4
Antidepressants	6062 (23.6)	1214 (26.7)	10.3	0.7	2846 (17.6)	−21.1	−2.9	391 (31.4)	25.0	0.2
Antihypertensives	1708 (6.6)	386 (8.5)	10.0	0.4	826 (5.1)	−9.3	−0.7	125 (10.1)	17.5	−0.1
Anxiolytics	460 (1.8)	87 (1.9)	1.4	−1.1	184 (1.1)	−7.7	−1.8	36 (2.9)	10.3	0
Barbiturates	1627 (6.3)	276 (6.1)	−1.5	1.4	829 (5.1)	−7.4	−2.7	102 (8.2)	10.2	−0.1
Benzodiazepines	3727 (14.5)	875 (19.3)	18.0	−6.5	1045 (6.5)	−37.5	−0.4	223 (17.9)	13.2	0.1
Mood stabilizers	813 (3.2)	203 (4.5)	9.7	1.4	337 (2.1)	−9.6	−0.9	43 (3.5)	2.4	−0.7
Other hypnotics	2783 (10.8)	609 (13.4)	11.2	3.8	1230 (7.6)	−15.8	−0.3	163 (13.1)	9.9	0.8
Stimulants for ADHD	548 (2.1)	161 (3.6)	12.1	2.4	152 (0.9)	−13.7	1.0	40 (3.2)	9.6	−0.3
Triptans	704 (2.7)	137 (3.0)	2.4	0.9	332 (2.1)	−6.4	−1.2	43 (3.5)	5.9	0.5
High utilization										
>120 MME for >90 d	16 843 (65.5)	3282 (72.3)	20.8	3.6	7638 (47.1)	−53.3	1.3	1083 (87.1)	74.1	0.4
>3 Opioid prescribers	2985 (11.6)	747 (16.5)	19.8	1.7	834 (5.2)	−33.2	0.5	218 (17.5)	23.8	−0.4

Abbreviations: ADHD, attention-deficit/hyperactivity disorder; MME, morphine milligram equivalent; NA, not applicable; SNRI, serotonin and norepinephrine reuptake inhibitor; SSRI, selective serotonin reuptake inhibitor.

^a^ Crude and adjusted standardized differences were based on fine stratification of an exposure propensity score. Variables with a weighted standardized difference greater than 10 were also included in outcome models.

^b^ Race and ethnicity data were obtained from Medicaid enrollment files.

^c^ Details on other category were not available.

### Statistical Analysis

We calculated the absolute risk of NOWS. Next, we calculated the relative risk (RR) of NOWS as follows: (1) unadjusted; (2) adjusted for confounding variables; (3) adjusted for opioid characteristics, including cumulative exposure, timing, and duration of exposure; and (4) adjusted for both opioid characteristics and confounding variables. Adjustment for opioid characteristics was performed to produce a comparable exposure across study medications given the differences in clinical use of the opioids of interest. Each adjusted model was based on fine stratification of an exposure propensity score that included cumulative exposure as a continuous term incorporated as a natural cubic spline. Other variables were treated as binary or categorical. The population was trimmed to the area of overlap, and 20 strata of the propensity score were defined according to the distribution of the exposed group. A stratum-specific weight was then assigned to the reference group to align the distributions of both populations for comparison.^23^ Variables with a standardized difference of 10% or greater after this adjustment were also included in the outcome model (as feasible) for further adjustment.

Several sensitivity analyses were performed. First, we evaluated subgroups by days’ supply in the 90 days before delivery (1-9, 10-29, or 30-90 days covered based on dispensing date and days’ supply for observed opioid prescriptions) and by quartile of cumulative exposure in MMEs. For this analysis, quartile cut points were selected on the basis of the distribution observed in the exposed group and applied to both the exposed and reference groups. Second, we assessed whether estimates varied if the most recent opioid prescription dispensing occurred within 1 to 29 days before delivery vs 30 to 90 days before delivery. Third, the outcome definition was restricted to include only severe NOWS. Because conversion factors used in calculating MMEs for particular opioids vary by source, we sought to quantify the extent to which the results were sensitive to the conversion factors used to calculate MMEs, and we applied a 50% discount and a 50% increase to the conversion factor for the exposure of interest without modifying the MME calculation for the reference group. Fourth, we used a 1:1 greedy match as an alternative to stratification and weighting to assess the extent to which the choice of adjustment method altered the study results.

When assessing each subgroup in these sensitivity analyses, the propensity score was recalculated and fine stratification and weighting were repeated. When the outcome model could not be fully adjusted for all covariates that retained an absolute standardized difference greater than 10 (designated in the footnotes to the applicable eTables 3 to 10 in the Supplement) because of limitations of model convergence, we used a manual stepwise approach to select the variables to include as a supplement to propensity score stratification and weighting.

Because the study focused on characterization of associations rather than statistical hypothesis testing, no a priori levels of significance were prespecified. All analyses were performed with SAS, version 9.4 (SAS Institute Inc), from February 2020 to March 2021.

## Results

Of the 80 903 eligible pregnancies in the MAX that were linked to live-born neonates and had 2 or more dispensed opioid prescriptions during the 90 days before delivery, 2412 (3.0%) were excluded because of presumed maternal opioid use disorder and 2787 (3.6%) were excluded owing to missing or invalid data on cumulative exposure in MMEs. Of the 75 704 remaining eligible pregnancies, 27 458 (36.3%) had prenatal exposure to more than 1 different type of opioid during the 90 days before delivery and 44 (0.1%) had prenatal exposure to an opioid monotherapy, which was observed for fewer than 50 patients. As a result, the cohort comprised 48 202 opioid-exposed pregnancies that met all inclusion criteria (Figure 1). A total of 1069 neonates (2.2%) born to these mothers had NOWS and 559 (1.2%) had severe NOWS.

Comparisons of opioid exposure during pregnancy were made between 25 710 pregnancies with hydrocodone exposure and 16 202 with codeine, 4540 with oxycodone, 1244 with tramadol, 260 with methadone, 93 with meperidine, 90 with hydromorphone, and 63 with morphine exposure. Opioid exposure characteristics varied substantially, with longer duration and higher cumulative exposure for oxycodone, hydromorphone, tramadol, methadone, and morphine compared with hydrocodone, codeine, and meperidine. Abdominal pain, arthritis or arthropathies, and back and neck pain were among the most common diagnosed pain conditions observed across all opioids. Migraine was also common, especially for meperidine (34.4%) and hydromorphone (27.8%) (Table; eTable 1 in the Supplement). Mothers for whom methadone was dispensed had higher rates of neuropathy or neuralgia (15.0%) than those who received dispensed hydrocodone (6.6%) (eTable 1 in the Supplement).

Demographic characteristics of mothers varied across the active ingredients of their opioid prescription. For example, a lower proportion of mothers with dispensed hydrocodone prescription (5.5%) lived in the northeastern US, compared with those with dispensed opioid with other active ingredients (oxycodone [25.6%], methadone [17.7%], codeine [10.1%], tramadol [11.8%]). Prescription of tramadol, oxycodone, methadone, hydromorphone, and morphine was more common among older mothers (≥35 years), and codeine and meperidine were less often prescribed in recent years (2010-2014) (Table; eTable 1 in the Supplement). Lower maternal comorbidity index scores were seen for mothers with hydrocodone, codeine, and tramadol dispensing, and higher values were seen for those with a methadone, hydromorphone, and morphine dispensing. A comparison of the demographic and clinical characteristics of those for whom hydrocodone was dispensed by NOWS status is shown in eTable 2 in the Supplement.

**Figure 2.**
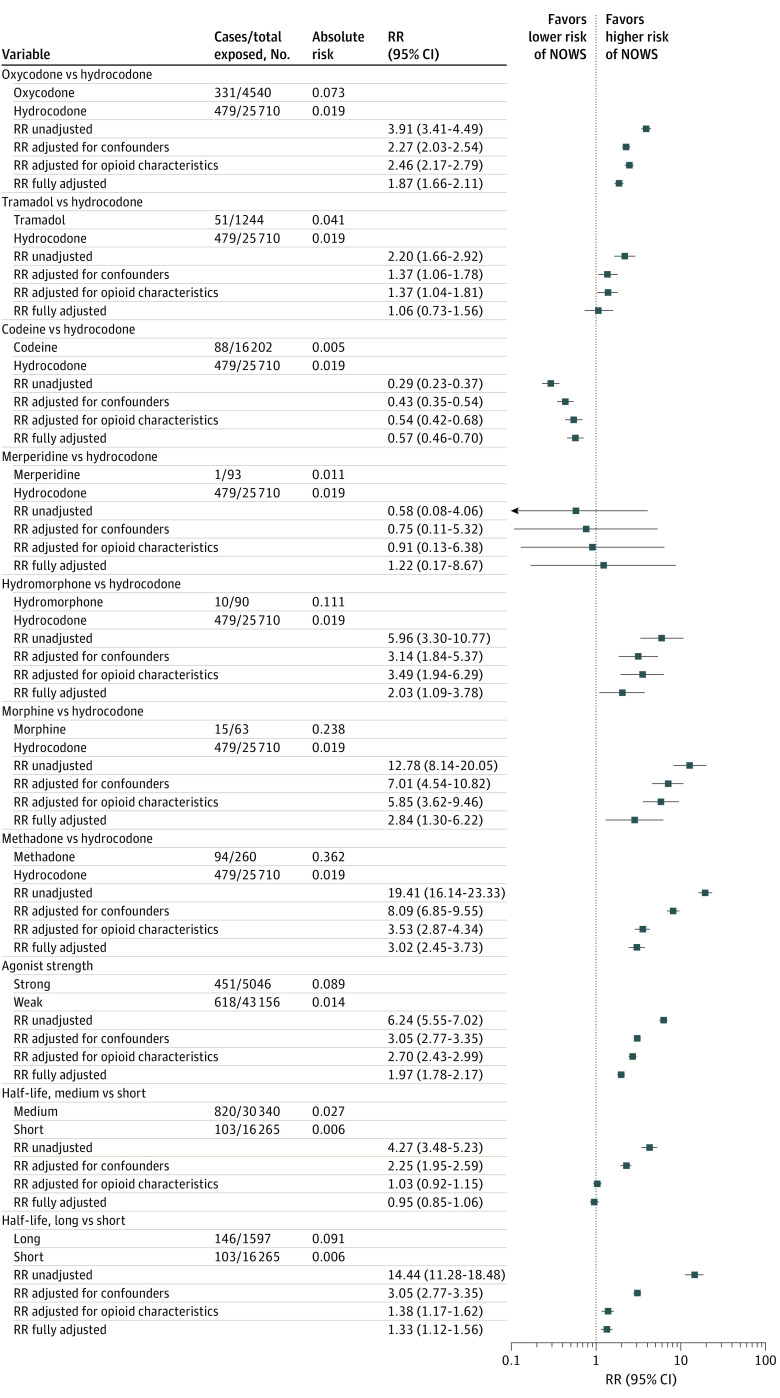
Association Between Opioid Medication Type and Neonatal Opioid Withdrawal Syndrome Risk Horizontal lines represent 95% CIs. RR indicates relative risk.

**Figure 3.**
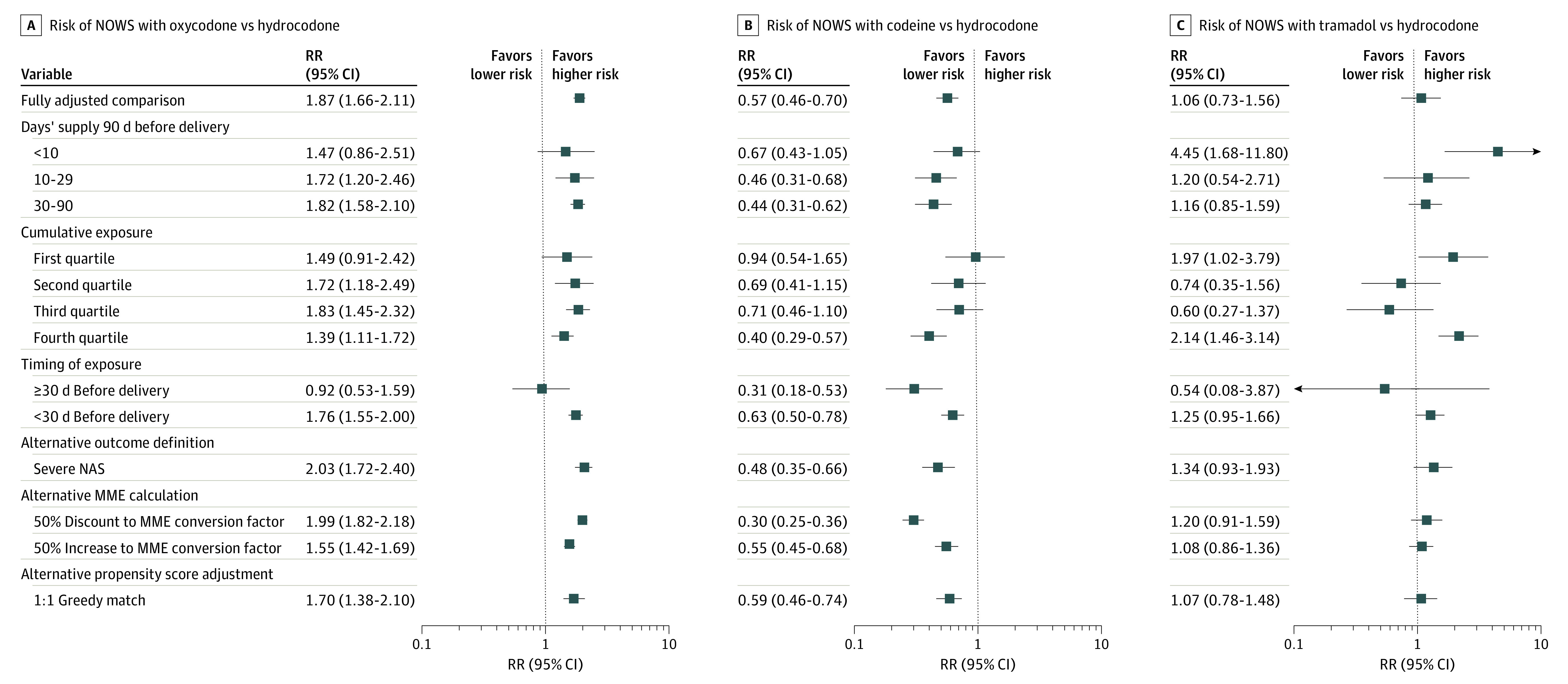
Sensitivity Analyses by Active Ingredient Horizontal lines represent 95% CIs. MME indicates morphine milligram equivalent; NAS, neonatal abstinence syndrome; NOWS, neonatal opioid withdrawal syndrome; RR, relative risk.

Unadjusted RR estimates suggested an increased risk of NOWS associated with most opioid medication types when compared against hydrocodone, ranging from 2.20 (95% CI, 1.66-2.92) for tramadol to 19.41 (95% CI, 16.14-23.33) for methadone. Exceptions were codeine (0.29; 95% CI, 0.23-0.37) and meperidine (0.58; 95% CI, 0.08-4.06) (Figure 2). All associations were substantially attenuated when adjusted for confounders and/or medication characteristics. In fully adjusted models that compared other opioid types against hydrocodone, codeine was associated with a lower risk of NOWS (RR, 0.57; 95% CI, 0.46-0.70), and risk of NOWS was not substantially different for tramadol (1.06; 95% CI, 0.73-1.56) (Figure 3). Increased risk was observed for oxycodone (1.87; 95% CI, 1.66-2.11), hydromorphone (2.03; 95% CI, 1.09-3.78), morphine (2.84; 95% CI, 1.30-6.22), and methadone (3.02; 95% CI, 2.45-3.73). Comparisons of meperidine vs hydrocodone (1.22; 95% CI, 0.17-8.67) had estimates with CIs that were too wide for meaningful interpretation (Figure 2).

Sensitivity analyses for the most common opioids are shown in Figure 3 and eTables 3 to 5 in the Supplement. For oxycodone, we did not observe substantial differences in RR estimates when assessing subgroups defined by days’ supply or cumulative exposure quartile in MMEs. Results were also comparable when adjusting for propensity score using a matched approach rather than a stratification and weighting approach. The risk of NOWS was comparable for oxycodone and hydrocodone when exposure occurred 30 days or more before delivery and was elevated only when exposure occurred within 30 days before delivery. Modification of the MME conversion factor did not meaningfully change interpretation. Moreover, the RR of severe NOWS was slightly more pronounced in the same direction as in the main analyses for all exposures. Mothers with dispensed codeine prescriptions were consistently at lower risk of NOWS except for the lowest quartile of cumulative exposure, wherein there was no meaningful difference between those with dispensed codeine and hydrocodone prescriptions. For tramadol, there was some variation in risk across sensitivity analyses, but most estimates suggested small or no differences between tramadol and hydrocodone. Exceptions included subgroups with less than 10 days’ supply and the highest quartile of cumulative exposure in MMEs (Figure 3; eTable 5 in the Supplement). Although small sample size resulted in imprecise estimates of effect for the less commonly prescribed opioids, all showed consistently elevated RR across sensitivity analyses (eTables 6 and 7 in the Supplement).

**Figure 4.**
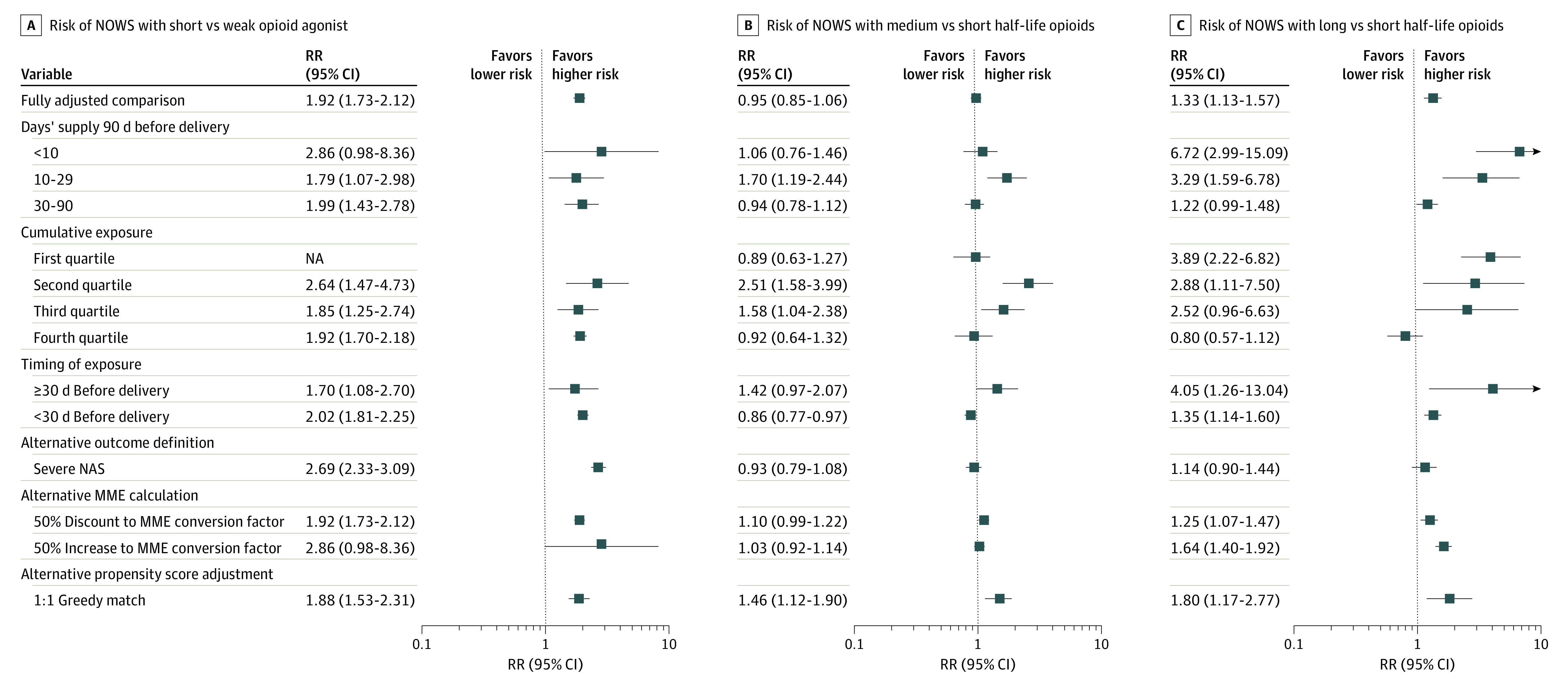
Sensitivity Analyses by Agonist Strength and Half-life Horizontal lines represent 95% CIs. MME indicates morphine milligram equivalent; NA, not applicable; NAS, neonatal abstinence syndrome; NOWS, neonatal opioid withdrawal syndrome; RR, relative risk.

In comparing strong vs weak opioid agonists, we found that neonates born to mothers who received strong agonists had a higher risk of NOWS, which persisted after adjustment for confounders and medication characteristics (RR, 1.97; 95% CI, 1.78-2.17). Unadjusted differences in risk of NOWS when comparing opioids by half-life were almost fully explained by characteristics of medication use. In fully adjusted comparisons of medium vs short half-life opioids, medium half-life products had an RR of 0.95 (95% CI, 0.85-1.06). In fully adjusted comparisons of long vs short half-life opioids, long half-life opioids were associated with an increased risk of NOWS (RR, 1.33; 95% CI, 1.12-1.56) (Figure 2). Sensitivity analyses showed consistently elevated risk of NOWS for neonates born to mothers who received strong agonists, but some variation in estimates by half-life was found (Figure 4; eTables 8 to 10 in the Supplement).

## Discussion

In this study, we observed differences in the risk of NOWS and severe NOWS among neonates with in utero exposure to opioid analgesics during the 90 days before birth; these differences were associated with the type of opioid prescribed, independent of differences in MMEs and other confounding factors. The risk was higher for newborns whose mothers received strong vs weak agonists; neonates with in utero exposure to oxycodone, methadone, hydromorphone, or morphine appeared to be at higher risk than those exposed to hydrocodone or codeine. Findings were consistent across various sensitivity and subgroup analyses and were robust to changes in conversion factors used in the calculation of cumulative exposure in MMEs. To our knowledge, this study was the first to compare the risk of NOWS by different types of prescription opioids for pain.

### Strengths and Limitations

This study has some strengths. It had a large sample size, used longitudinal data from multiple health care settings, gave attention to control of confounding, and prospectively collected exposure data to eliminate the potential for recall bias.

This study also has several limitations. First, data on medication exposure were captured from opioid prescription dispensing, which may imperfectly translate to opioid exposure. Medication could be purchased and saved for use at a later time, shared, or sold. Furthermore, opioids from nonmedical sources were not captured. Although such data were less likely to alter the study findings given that all mothers and neonates had known opioid treatment and mothers with opioid use disorders were excluded, some differences may remain among those with dispensed opioid medications in this analysis.

Second, although not indicated by current guidelines, we cannot exclude the possibility that screening for NOWS after delivery may be more intensive in mothers who received certain types of opioids. All mothers received 2 or more dispensed opioid prescriptions, and analyses were adjusted for cumulative exposure measured in MMEs in the last 90 days of pregnancy; however, it is possible that clinicians are more aware of the risk of NOWS for neonates born to mothers who received medications that are typically given at higher doses and for longer durations than hydrocodone or codeine. Although the analyses were adjusted for characteristics of opioid treatment course, surveillance bias could result if more sensitive capture of risk of NOWS for the strong agonist medications was performed, which would exaggerate the differences in risk.

Third, to assess NOWS, we necessarily restricted the population to mothers who could be linked to a live-born neonate. This linkage was possible only if a subscriber identification number had been assigned to a neonate, which was not likely if the newborn died shortly after delivery. If early death was more common in neonates born to mothers with exposure to certain opioids, selection bias could result. However, to our knowledge, no evidence suggests that there are differences in the frequency of perinatal death associated with the type of opioid exposure. Furthermore, given the rarity of nonlive birth, even if such a difference existed, its role in the risk estimates from this study is expected to be minor.

Fourth, data were available only through 2014 because of lag time in the availability of new information from the Centers for Medicaid & Medicare Services. Both incidence of NOWS and health care professional practices concerning opioid use during pregnancy have changed over time; however, one would not expect a difference in the underlying biological association between opioid properties and NOWS.

Fifth, although we adjusted for a variety of confounders, not all were well measured using administrative claims sources. Furthermore, in the sensitivity analyses, full adjustment was not always possible owing to limited sample size within subgroups of interest. Given that the direction of confounding observed was away from the null and that observed confounding was severe in some instances, these results may overestimate the differences between hydrocodone and the less common strong agonists within the affected subgroup.

## Conclusions

Assessing opioids as a class may mask important differences between medications that are relevant to clinical decision-making. In this cohort study, we observed higher risks of NOWS and severe NOWS in neonates born to mothers for whom oxycodone, methadone, morphine, and hydromorphone prescriptions were dispensed compared with neonates born to mothers who had similar cumulative exposure to hydrocodone. Although pain management needs vary substantially across patients, information on opioid-specific risks of NOWS may help prescribers select an opioid to treat pain in late stages of pregnancy.

## Notes

Supplement. **eTable 1.** All Potential Covariates Considered for Inclusion in Adjusted Models **eTable 2.** Characteristics of Hydrocodone Users by NOWS Status **eTable 3.** Association Between Oxycodone and NOWS **eTable 4.** Association Between Codeine and NOWS **eTable 5.** Association Between Tramadol and NOWS **eTable 6.** Association Between Methadone and NOWS **eTable 7.** Association Between Morphine and NOWS **eTable 8.** Association Between Agonist Strength and NOWS **eTable 9.** Association Between Opioid Half-Life (Medium vs. Short) and NOWS **eTable 10.** Association Between Opioid Half-Life (Long vs. Short) and NOWS
