# Supplementary material for: Characteristics of Prescription Opioid Analgesics in Pregnancy and Risk of Neonatal Opioid Withdrawal Syndrome in Newborns
Source: JAMA Netw Open. 2022 Aug 24;5(8):e2228588. doi: 10.1001/jamanetworkopen.2022.28588 (PMC9403776; doi:10.1001/jamanetworkopen.2022.28588)
Supplement: Supplement. — eTable 1. All Potential Covariates Considered for Inclusion in Adjusted Models eTable 2. Characteristics of Hydrocodone Users by NOWS Status eTable 3. Association Between Oxycodone and NOWS eTable 4. Association Between Codeine and NOWS eTable 5. Association Between Tramadol and NOWS eTable 6. Association Between Methadone and NOWS eTable 7. Association Between Morphine and NOWS eTable 8. Association Between Agonist Strength and NOWS eTable 9. Association Between Opioid Half-Life (Medium vs. Short) and NOWS eTable 10. Association Between Opioid Half-Life (Long vs. Short) and NOWS [file jamanetwopen-e2228588-s001.pdf]

Supplemental Online Content

Esposito DB, Huybrechts KF, Werler MM, et al. Characteristics of prescription opioid analgesics in pregnancy and risk of neonatal opioid withdrawal syndrome in newborns. *JAMA Netw Open*. 2022;5(8):e2228588. doi:10.1001/jamanetworkopen.2022.28588

eTable 1. All Potential Covariates Considered for Inclusion in Adjusted Models

eTable 2. Characteristics of Hydrocodone Users by NOWS Status

eTable 3. Association Between Oxycodone and NOWS

eTable 4. Association Between Codeine and NOWS

eTable 5. Association Between Tramadol and NOWS

eTable 6. Association Between Methadone and NOWS

eTable 7. Association Between Morphine and NOWS

eTable 8. Association Between Agonist Strength and NOWS

eTable 9. Association Between Opioid Half-Life (Medium vs. Short) and NOWS

eTable 10. Association Between Opioid Half-Life (Long vs. Short) and NOWS

This supplemental material has been provided by the authors to give readers additional information about their work.

© 2022 Esposito DB et al. *JAMA Network Open.*

Supplement 1: All potential covariates considered for inclusion in adjusted models

	Patient characteristics by opioid monotherapy drug																						
	Hydrocodone		Oxycodone			Methadone			Codeine			Tramadol			Meperidine			Hydromorphone			Morphine		
	N	%	N	%	SD	N	%	SD	N	%	SD	N	%	SD	N	%	SD	N	%	SD	N	%	SD
**All**	**25,710**	**100.0**	**4,540**	**100.0**		**260**	**100.0**		**16,202**	**100.0**		**1,244**	**100.0**		**93**	**100.0**		**90**	**100.0**		**63**	**100.0**	
**Region**																							
West	6,024	23.4	579	12.8	-39.6	45	17.3	-21.6	2781	17.2	-22.1	196	15.8	-27.5	13	14.0	-34.5	*		-51.8	13	20.6	-9.6
Central	8,384	32.6	1,366	30.1	-7.7	89	34.2	4.9	7,107	43.9	33.0	443	35.6	9.0	24	25.8	-21.2	35	38.9	18.6	26	41.3	25.5
South	9,895	38.5	1,433	31.6	-20.6	80	30.8	-23.0	4,686	28.9	-28.8	458	36.8	-4.9	48	51.6	37.6	23	25.6	-39.6	15	23.8	-45.4
Northeast	1,407	5.5	1,162	25.6	81.8	46	17.7	55.0	1,628	10.1	24.3	147	11.8	32.2	*		17.3	23	25.6	81.7	*		42.3
**Age category**																							
<18	732	2.9	76	1.7	-11.2	*		-27.9	617	3.8	7.6	20	1.6	-11.9	*		18.1	0	0.0	-34.3	0	0.0	-34.3
18-24	11,103	43.2	1,574	34.7	-24.8	55	21.2	-68.7	7,733	47.7	12.9	400	32.2	-32.4	45	48.4	14.8	35	38.9	-12.4	14	22.2	-64.9
25-34	12,046	46.9	2,420	53.3	18.3	171	65.8	55.0	6,890	42.5	-12.3	678	54.5	21.7	35	37.6	-26.5	47	52.2	15.2	36	57.1	29.3
35+	1,829	7.1	470	10.4	16.3	33	12.7	26.5	962	5.9	-6.7	146	11.7	22.5	*		7.8	*		9.3	13	20.6	56.4
**Year of delivery**																							
2000-2004	4,254	16.6	602	13.3	-13.1	27	10.4	-25.7	5495	33.9	57.7	142	11.4	-21.0	47	50.5	109.1	*		-38.3	*		-2.6
2005-2009	11,120	43.3	1,671	36.8	-18.6	127	48.9	15.9	6,545	40.4	-8.2	442	35.5	-22.4	31	33.3	-29.0	38	42.2	-2.9	29	46.0	7.9
2010-2014	10,336	40.2	2,267	49.9	27.8	106	40.8	1.6	4,162	25.7	-44.2	660	53.1	36.7	15	16.1	-78.5	45	50.0	28.0	24	38.1	-6.1
**Race/ethnicity**																							
White	18,725	72.8	3,039	66.9	-18.2	217	83.5	36.7	9,581	59.1	-41.3	913	73.4	1.8	56	60.2	-38.1	64	71.1	-5.4	42	66.7	-19.0
Black	3,777	14.7	888	19.6	18.3	13	5.0	-46.6	4210	26.0	40.1	158	12.7	-8.2	24	25.8	39.5	16	17.8	11.9	12	19.1	16.5
Hispanic/Latinx	1,448	5.6	185	4.1	-10.3	*		-14.8	1222	7.5	10.9	74	6.0	1.9	*		4.9	*		-7.7	*		4.3
Other	1,760	6.9	428	9.4	13.4	21	8.1	6.6	1189	7.3	2.7	99	8.0	6.0	*		3.7	*		-1.0	*		5.9
**Infant sex**																							
Male	12,226	47.6	2,143	47.2	-1.0	114	43.9	-10.5	7,708	47.6	0.1	605	48.6	3.1	44	47.3	-0.7	44	48.9	3.8	29	46.0	-4.3
Male/Female twins	567	2.2	105	2.3	1.0	*		1.0	431	2.7	4.1	20	1.6	-6.2	*	1.1	-12.6	*		17.6	*		-6.4
Female	12,850	50.0	2,289	50.4	1.2	140	53.9	11.0	8,010	49.4	-1.5	616	49.5	-1.3	48	51.6	4.6	42	46.7	-9.4	33	52.4	6.8
Other/unknown	67	0.3	*		-6.6	0	0.0	-10.2	53	0.3	1.8	*		-0.6	0	0.0	-10.2	0	0.0	-10.2	0	0.0	-10.2
**Multiparous**	19,707	76.7	3,547	78.1	5.0	215	82.7	21.3	12,102	74.7	-6.5	999	80.3	12.6	61	65.6	-34.8	61	67.8	-28.1	53	84.1	26.8
**Multifetal gestation**	573	2.2	107	2.4	1.2	*		0.8	397	2.5	2.1	16	1.3	-10.1	*		8.7	*		9.5	*		8.2
**Tobacco use**	2,585	10.1	543	12.0	8.6	31	11.9	8.5	1021	6.3	-19.4	145	11.7	7.3	*		-18.5	12	13.3	14.5	*		-2.5
Current use	2,515	9.8	532	11.7	8.9	30	11.5	8.1	1012	6.3	-18.4	143	11.5	7.9	*		-17.3	12	13.3	15.7	*		-1.3
Cessation treatment	157	0.6	34	0.8	2.4	*		-4.6	24	0.2	-10.6			-2.5	*		7.3	0	0.0	-15.7	0	0.0	-15.7
History of use	129	0.5	31	0.7	3.3	0	0.0	-14.2	45	0.3	-5.0	*		-4.0	0	0.0	-14.2	*		29.4	0	0.0	-14.2
**Maternal Morbidity Index**																							
0	10,920	42.5	1,455	32.1	-30.7	41	15.8	-87.0	7,707	47.6	14.5	488	39.2	-9.3	31	33.3	-26.8	13	14.4	-92.4	*		-107.1
1	6,591	25.6	1,070	23.6	-6.8	28	10.8	-55.5	4,104	25.3	-1.0	319	25.6	0.0	29	31.2	17.4	16	17.8	-27.1	23	36.5	33.5
2	3,999	15.6	851	18.7	12.0	84	32.3	56.7	2230	13.8	-7.2	203	16.3	3.0	*		-20.1	23	25.6	35.3	*		-5.0
3+	4,200	16.3	1,164	25.6	32.5	107	41.2	80.6	2,161	13.3	-12.0	234	18.8	9.2	23	24.7	29.5	38	42.2	83.9	24	38.1	71.3
**Potential opioid indications**																							
Abdominal pain	9,437	36.7	1,883	41.5	13.8	73	28.1	-26.2	5,635	34.8	-5.7	362	29.1	-23.0	43	46.2	27.5	41	45.6	25.5	21	33.3	-10.0
Arthritis arthropathies	5,249	20.4	1,072	23.6	10.9	49	18.9	-5.6	2,334	14.4	-22.5	298	24.0	12.0	19	20.4	0.0	17	18.9	-5.5	14	22.2	6.2
Back and neck pain	8,444	32.8	1,910	42.1	27.1	119	45.8	37.8	3,277	20.2	-40.8	498	40.0	21.2	24	25.8	-21.9	32	35.6	8.1	29	46.0	38.5
Complex regional pain syndrome	18	0.1	16	0.4	8.7	*		9.3	*		-4.2	*		3.8	0	0.0	-5.3	0	0.0	-5.3	0	0.0	-5.3
Cough	1,356	5.3	253	5.6	1.9	11	4.2	-6.9	669	4.1	-7.6	41	3.3	-13.8	*		-23.4	*		1.8	*		-3.3
Crohn's disease	64	0.3	28	0.6	8.0	*		3.3	23	0.1	-3.5	*		-0.3	0	0.0	-10.0	*		14.8	0	0.0	-10.0
Dental	2,460	9.6	360	7.9	-8.2	19	7.3	-11.5	1237	7.6	-9.8	151	12.1	11.7	*		-36.9	*		-21.5	*		-37.4
Fibromyalgia	763	3.0	247	5.4	17.4	28	10.8	44.1	246	1.5	-13.9	70	5.6	18.6	*		2.1	*		2.9	*		22.8
Generalized pain	109	0.4	48	1.1	10.6	0	0.0	-13.0	52	0.3	-2.3	10	0.8	6.9	0	0.0	-13.0	*		22.4	*		16.6
Infection	2,387	9.3	476	10.5	5.7	18	6.9	-12.2	1604	9.9	3.0	120	9.7	1.8	11	11.8	11.8	13	14.4	22.6	*		22.0
Joint pain	2,543	9.9	507	11.2	5.9	22	8.5	-7.0	1042	6.4	-17.9	174	14.0	17.9	*		-24.1	*		-30.1	*		19.1
Lupus	21	0.1	12	0.3	6.2	0	0.0	-5.7	*		-1.7	*		0.0	0	0.0	-5.7	*		19.0	0	0.0	-5.7
Malignancy	81	0.3	23	0.5	4.2	*		13.8	63	0.4	1.7	*		6.6	0	0.0	-11.3	*		24.1	*		18.5
Migraine headache	5,074	19.7	883	19.5	-1.0	21	8.1	-48.3	2634	16.3	-12.8	211	17.0	-10.2	32	34.4	47.3	25	27.8	26.8	*		-20.6
Orthopedic injury	3,555	13.8	655	14.4	2.4	25	9.6	-18.6	1678	10.4	-15.1	176	14.2	1.3	12	12.9	-3.9	*		-27.7	*		-26.9
Osteoarthritis	312	1.2	90	2.0	8.7	*		11.8	52	0.3	-14.5	34	2.7	15.5	*		-1.7	0	0.0	-22.1	*		4.6
Other neuropathies	1,684	6.6	514	11.3	23.7	39	15.0	38.9	510	3.2	-22.5	99	8.0	7.7	*		-7.0	*		6.8	*		42.2
Pain not elsewhere classified	889	3.5	312	6.9	21.9	34	13.1	50.2	145	0.9	-25.0	67	5.4	13.3	0	0.0	-37.9	*		26.6	*		9.3
Pain related to psychologic factors	25	0.1	17	0.4	7.9	*		8.1	*		-2.0	*		2.4	0	0.0	-6.3	0	0.0	-6.3	0		-6.3
Peripheral neuropathy	*		*		-0.9	0	0.0	-3.5	*		-2.0	*		3.0	0	0.0	-3.5	*		29.5	0		-3.5
Rheumatoid arthritis	90	0.4	25	0.6	4.2	*		0.7	31	0.2	-4.4	12	1.0	10.7	*		12.3	0	0.0	-11.9	0		-11.9
Sarcoidosis	15	0.1	*		-2.8	0	0.0	-4.9	*		-3.8	0	0.0	-4.9	0	0.0	-4.9	0	0.0	-4.9	*		24.0
Sciatica	508	2.0	121	2.7	6.5	*		9.9	164	1.0	-11.3	25	2.0	0.3	*		-10.4	0	0.0	-28.4	*		-4.2
Sickle cell disease	51	0.2	70	1.5	20.5	*		11.6	32	0.2	0.0	*		-1.3	*		15.6	*		60.3	*		56.5
Trigeminal neuralgia	14	0.1	*		5.4	0	0.0	-4.5	*		-3.3	*		1.7	0	0.0	-4.5	0	0.0	-4.5	0		-4.5
Ulcerative colitis	21	0.1	12	0.3	6.2	0	0.0	-5.7	11	0.1	-0.5	0	0.0	-5.7	0	0.0	-5.7	0	0.0	-5.7	0		-5.7
Urinary calculus	733	2.9	218	4.8	14.4	*	0.8	-22.1	267	1.7	-11.5	16	1.3	-15.5	*		35.3	16	17.8	71.6	*		23.7
Post-surgical pain	681	2.7	269	5.9	23.0	*	3.5	6.7	506	3.1	4.0	34	2.7	0.7	*		4.9	11	12.2	52.5	*		-10.4
Revised indication groups																							
Abdominal pain	9,437	36.7	1,883	41.5	13.8	73	28.1	-26.2	5,635	34.8	-5.7	362	29.1	-23.0	43	46.2	27.5	41	45.6	25.5	21	33.3	-10.0
Arthritis arthropathies	5423	21.1	1116	24.6	11.8	54	20.8	-1.1	2363	14.6	-24.1	320	25.7	15.5	19	20.4	-2.3	17	18.9	-7.8	15	23.8	9.2
Back and neck pain	8,444	32.8	1,910	42.1	27.1	119	45.8	37.8	3,277	20.2	-40.8	498	40.0	21.2	24	25.8	-21.9	32	35.6	8.1	29	46.0	38.5
Dental	2,460	9.6	360	7.9	-8.2	19	7.3	-11.5	1237	7.6	-9.8	151	12.1	11.7	*		-36.9	*		-21.5	*		-37.4
Infection	2,387	9.3	476	10.5	5.7	18	6.9	-12.2	1604	9.9	3.0	120	9.7	1.8	11	11.8	11.8	13	14.4	22.6	*		22.0
Joint pain	2,543	9.9	507	11.2	5.9	22	8.5	-7.0	1042	6.4	-17.9	174	14.0	17.9	*		-24.1	*		-30.1	*		19.1
Migraine headache	5,074	19.7	883	19.5	-1.0	21	8.1	-48.3	2634	16.3	-12.8	211	17.0	-10.2	32	34.4	47.3	25	27.8	26.8	*		-20.6
Orthopedic injury	3,555	13.8	655	14.4	2.4	25	9.6	-18.6	1678	10.4	-15.1	176	14.2	1.3	12	12.9	-3.9	*		-27.7	*		-26.9
Neuropathy/neuralgias	1699	6.6	520	11.5	24.0	39	15.0	38.6	511	3.2	-22.8	100	8.0	7.8	*		-7.3	*		6.4	*		41.9
Autoimmune pain	112	0.4	49	1.1	10.5	*		-1.1	42	0.3	-4.2	*		-2.6	0	0.0	-13.2	*		22.1	*		16.3
Other chronic pain	1016	4.0	392	8.6	27.4	33	12.7	45.3	394	2.4	-12.2	88	7.1	19.4	*		2.5	12	13.3	47.9	*		57.6
Other acute pain	3410	13.3	942	20.7	28.3	53	20.4	27.0	1511	9.3	-17.6	142	11.4	-8.0	13	14.0	3.0	30	33.3	69.1	*		10.5
**Psychiatric conditions**	5555	21.6	1223	26.9	17.6	87	33.5	37.9	2572	15.9	-20.8	336	27.0	17.9	19	20.4	-4.1	22	24.4	9.5	18	28.6	22.8
ADHD	318	1.2	79	1.7	5.8	*		3.6	109	0.7	-8.3	34	2.7	15.1	*		-2.1	*		-1.7	*		4.2
Anxiety	2,591	10.1	539	11.9	8.1	43	16.5	27.0	939	5.8	-22.5	154	12.4	10.3	*		-18.7	12	13.3	14.3	*		18.2
Bipolar disorder	968	3.8	254	5.6	12.2	16	6.2	15.5	474	2.9	-6.6	60	4.8	7.3	*		-4.2	*		-3.4	*		6.9
Depression	2,889	11.2	646	14.2	12.7	49	18.9	30.3	1539	9.5	-8.1	162	13.0	7.7	*		-12.5	13	14.4	13.5	12	19.1	31.0
Posttraumatic stress disorder	315	1.2	99	2.2	10.4	*		14.9	162	1.0	-3.1	17	1.4	1.8	0	0.0	-22.3	*		20.0	0	0.0	-22.3
Schizophrenia	55	0.2	15	0.3	3.3		0.0	-9.2	38	0.2	0.6	*		8.0	*		15.4	0	0.0	-9.2	0	0.0	-9.2
Sleep disorders	495	1.9	121	2.7	7.0	11	4.2	18.9	173	1.1	-10.0	37	3.0	9.5	*		2.2	*		12.4	*		11.1
**Substance use**	645	2.5	155	3.4	7.6	*		4.9	383	2.4	-1.3	48	3.9	10.9	*		6.1	*		6.9	*		17.1
Alcohol abuse dependence	239	0.9	80	1.8	10.2	*		11.8	163	1.0	1.2	25	2.0	12.7	*		2.1			14.7	0	0.0	-19.4
Amphetamine use disorder	103	0.4	11	0.2	-4.0	*		6.9	58	0.4	-0.9	*		7.3	0	0.0	-12.7	0	0.0	-12.7	0	0.0	-12.7
Cocaine use	98	0.4	33	0.7	6.7	*		7.3	58	0.4	-0.5	*		5.2	*		11.6	*		12.0	0	0.0	-12.4
Hallucinogen use	*			0.0	-2.0	0	0.0	-2.0	0	0.0	-2.0	0		-2.0	0	0.0	-2.0	0	0.0	-2.0	0	0.0	-2.0
Marijuana use	263	1.0	53	1.2	2.0	*		-10.9	154	1.0	-1.0	14	1.1	1.5	*		12.8	0	0.0	-20.3	*		7.1
Sedative hypnotic use disorders	21	0.1	*		2.9	0	0.0	-5.7	*		-1.1	*		7.6	0	0.0	-5.7	0	0.0	-5.7	*		34.8
**Other comorbidities**																							
Anemia	608	2.4	197	4.3	15.6	*		12.2	374	2.3	-0.5	26	2.1	-2.6	*		15.3	13	14.4	63.1	*		68.3
Antiphospholipid syndrome	*		*		5.0	0	0.0	-2.8	*		0.9	0	0.0	-2.8	0	0.0	-2.8			20.6	0	0.0	-2.8
Chronic pulmonary dis	2,651	10.3	541	11.9	7.3	29	11.2	3.8	1487	9.2	-5.4	131	10.5	1.0	*		2.0	11	12.2	8.6	*	15.9	23.4
Diabetes	794	3.1	183	4.0	7.2	*		-6.8	457	2.8	-2.3	46	3.7	4.8	*		-19.9	*		10.0	*		37.7
Gout	12	0.1	*		1.2	0	0.0	-4.5	*		-0.7	*		1.7	0	0.0	-4.5	0		-4.5	0	0.0	-4.5
Hypertension	1,240	4.8	304	6.7	11.4	14	5.4	3.6	598	3.7	-7.9	84	6.8	11.7	*		3.6	*		33.1	*		9.4
Infertility	44	0.2	*		0.3	0	0.0	-8.3	46	0.3	3.3	*		6.1	0	0.0	-8.3	*		16.7	*		33.3
Obesity overweight	1,016	4.0	260	5.7	11.7	*		-13.3	447	2.8	-9.4	47	3.8	-1.3	*		9.6	*		3.5	*		45.4
Polycystic ovarian syndrome	58	0.2	14	0.3	2.2	0	0.0	-9.6	35	0.2	-0.3	*		2.4	*		14.9	*		15.3	0	0.0	-9.6
Renal disease	61	0.2	18	0.4	4.0	0	0.0	-9.8	22	0.1	-3.3	*		8.6	0	0.0	-9.8	*		15.1	*		20.1
Thyroid disorders	744	2.9	154	3.4	4.1	13	5.0	15.4	411	2.5	-3.1	28	2.3	-5.7	*		2.8	*		3.6	*		2.3
**Medication use**																							
**Medications during the 90 days before delivery**																							
Atypical antipsychotics	36	0.1	*		2.1	*		6.7	21	0.1	-0.4	*		10.1	0	0.0	-7.5	0	0.0	-7.5	0	0.0	-7.5
Barbiturates	808	3.1	166	3.7	4.1	*		-3.8	554	3.4	2.2	64	5.1	14.2	*		15.7	*		-8.1	0	0.0	-36.0
Benzodiazepines, long acting	290	1.1	77	1.7	6.8	*		5.1	55	0.3	-13.1	14	1.1	0.0	*		11.4	0	0.0	-21.4	*		5.6
Benzodiazepines, short acting	1,634	6.4	428	9.4	16.1	42	16.2	44.3	324	2.0	-31.0	111	8.9	13.6	0	0.0	-52.1	*		-29.1	*		-9.9
SNRI	234	0.9	53	1.2	3.6	*		22.0	81	0.5	-6.9	33	2.7	18.7	0	0.0	-19.2	*		15.0	*		8.7
SSRI	2,551	9.9	477	10.5	2.8	31	11.9	9.1	1297	8.0	-9.5	147	11.8	8.6	*		-6.4	12	13.3	15.1	*		-9.8
Tricyclic antidepressants	231	0.9	70	1.5	8.3	*		3.5	76	0.5	-7.4	28	2.3	15.4	*		30.4	*		3.0	*		22.8
Other antidepressants	602	2.3	146	3.2	7.6	16	6.2	26.9	289	1.8	-5.6	37	3.0	5.5	*		22.4	*		-1.1	*	3.2	7.2
Other hypnotics	3,349	13.0	735	16.2	12.7	36	13.9	3.4	1736	10.7	-10.2	171	13.8	3.0	21	22.6	35.6	19	21.1	30.6	*		11.4
**Medications during the baseline period**																							
Anti Nausea	11,162	43.4	1,897	41.8	-4.7	111	42.7	-2.1	5,426	33.5	-29.0	492	39.6	-11.1	56	60.2	48.2	54	60.0	47.6	18	28.6	-44.3
Antibiotics	17,211	66.9	2,979	65.6	-4.0	156	60.0	-20.4	10,671	65.9	-3.2	791	63.6	-10.0	69	74.2	22.6	63	70.0	9.3	35	55.6	-33.3
Anticonvulsants	1,692	6.6	495	10.9	21.7	40	15.4	40.2	583	3.6	-19.2	147	11.8	25.8	*		-0.7	*		22.6	*		29.5
Antidepressants	6,062	23.6	1,214	26.7	10.3	105	40.4	51.8	2,846	17.6	-21.1	391	31.4	25.0	21	22.6	-3.4	18	20.0	-12.3	19	30.2	21.1
Antidiabetic	358	1.4	70	1.5	1.8	*		1.8	164	1.0	-4.9	20	1.6	2.6	0	0.0	-23.8	0	0.0	-23.8	*		27.7
Antihypertensive	1,708	6.6	386	8.5	10.0	27	10.4	19.0	826	5.1	-9.3	125	10.1	17.5	*		15.7	*		22.3	*		7.1
Anxiolytics	460	1.8	87	1.9	1.4	*		-2.8	184	1.1	-7.7	36	2.9	10.3	0	0.0	-27.0	*		4.3	*		12.6
Atypical APM	64	0.3	19	0.4	4.2	*		3.3	29	0.2	-2.1	*		6.9	0	0.0	-10.0	0	0.0	-10.0	*		-10.0
Barbiturates	1,627	6.3	276	6.1	-1.5	*		-21.8	829	5.1	-7.4	102	8.2	10.2	11	11.8	27.2	*		2.0	*		-34.6
Benzodiazepines	3,727	14.5	875	19.3	18.0	84	32.3	60.9	1045	6.5	-37.5	223	17.9	13.2	*		-64.9	15	16.7	8.5	17	27.0	44.1

© 2022 Esposito DB et al. *JAMA Network Open.*

Corticosteroids	4,647	18.1	897	19.8	6.1	42	16.2	-7.2	2538	15.7	-9.1	263	21.1	10.9	17	18.3	0.8	20	22.2	14.7	16	25.4	25.2
Insulin	435	1.7	105	2.3	6.3	*		-6.5	298	1.8	1.6	24	1.9	2.6	*		-7.4	*		14.9	*		24.7
Mood Stabilizer	813	3.2	203	4.5	9.7	14	5.4	15.6	337	2.1	-9.6	43	3.5	2.4	*		-8.9	*		16.7	*		-14.6
Other hypnotics	2,783	10.8	609	13.4	11.2	44	16.9	25.1	1230	7.6	-15.8	163	13.1	9.9	12	12.9	9.1	13	14.4	15.4	*		-14.0
Progestins	954	3.7	170	3.7	0.2	*		-11.6	468	2.9	-6.5	48	3.9	1.1	*		-13.1	*		18.9	*		17.1
Stimulants for ADHD	548	2.1	161	3.6	12.1	18	6.9	32.8	152	0.9	-13.7	40	3.2	9.6	0	0.0	-29.5	*		-11.4	*		-5.7
Suspected Teratogens	3,918	15.2	751	16.5	5.0	26	10.0	-22.4	2230	13.8	-6.0	190	15.3	0.1	17	18.3	11.5	16	17.8	9.7	*		-3.8
Triptans	704	2.7	137	3.0	2.4	12	4.6	14.1	332	2.1	-6.4	43	3.5	5.9	*		19.0	*		4.9	*		24.6
Typical APM	758	3.0	185	4.1	8.6	14	5.4	17.2	331	2.0	-8.3	69	5.6	18.3	*		-18.9	*		24.7	*		31.3
**High utilization**																							
> 120 MME for >90 days	16,843	65.5	3,282	72.3	20.8	224	86.2	70.3	7,638	47.1	-53.3	1,083	87.1	74.1	53	57.0	-24.8	59	65.6	0.2	45	71.4	18.1
>3 opioid prescribers	2,985	11.6	747	16.5	19.8	57	21.9	39.4	834	5.2	-33.2	218	17.5	23.8	*		-31.8	14	15.6	16.3	*		4.7
**Infant/delivery characteristics**																							
Congenital Malformation	2,019	7.9	457	10.1	11.0	18	6.9	-5.0	1310	8.1	1.3	131	10.5	13.1	*		3.9	13	14.4	29.8	11	17.5	41.3
Chromosomal Anomaly	44	0.2	*		0.3				20	0.1	-1.9	*		-3.6									
Preterm	3,280	12.8	749	16.5	15.0	53	20.4	29.1	1769	10.9	-8.1	213	17.1	17.3	17	18.3	21.6	24	26.7	50.2	*		12.6
Preeclampsia	897	3.5	207	4.6	7.7	*		-3.3	556	3.4	-0.5	51	4.1	4.5	*		-2.0	*		26.4	*		-2.5
Placental Abruption	366	1.4	79	1.7	3.6	*		5.5	169	1.0	-4.9	17	1.4	-0.6	*		-4.3				*		2.0
IUGR SGA	1,161	4.5	239	5.3	4.9	31	11.9	38.5	572	3.5	-7.1	70	5.6	7.2	*		-1.5	*		13.2	*		41.7
**Opioid use**																							
**Total days supply**																							
**Days 90-61 before delivery**																							
0	9,173	35.7	1,407	31.0	-14.1	33	12.7	-78.8	7,501	46.3	30.7	272	21.9	-43.7	36	38.7	8.9	27	30.0	-17.1	14	22.2	-42.4
1 - 10	10,696	41.6	1,656	36.5	-14.9	57	21.9	-61.2	7,722	47.7	17.3	369	29.7	-35.5	44	47.3	16.3	41	45.6	11.3	15	23.8	-54.6
11 - 20	3,647	14.2	838	18.5	16.4	81	31.2	58.5	768	4.7	-46.3	338	27.2	45.9	12	12.9	-5.3	19	21.1	25.8	13	20.6	24.1
21 - 30	2,194	8.5	639	14.1	24.8	89	34.2	93.4	211	1.3	-48.0	265	21.3	51.5	*		-50.0	*		-31.3	21	33.3	90.5
**Days 60-31 before delivery**																							
0	6,063	23.6	853	18.8	-16.6	*		-92.0	5204	32.1	27.1	106	8.5	-59.3	22	23.7	0.3	30	33.3	30.7	*		-54.5
1 - 10	11,295	43.9	1,558	34.3	-28.0	18	6.9	-132.8	9,456	58.4	41.3	301	24.2	-60.2	43	46.2	6.6	25	27.8	-48.3	12	19.1	-78.6
11 - 20	3,260	12.7	603	13.3	2.5	36	13.9	4.9	1062	6.6	-29.6	256	20.6	30.2	21	22.6	37.1	14	15.6	11.7	*		-14.2
21 - 30	5,092	19.8	1,526	33.6	44.7	199	76.5	195.0	480	3.0	-77.8	581	46.7	84.2	*		-51.4	21	23.3	12.1	39	61.9	134.0
**Days 30-0 before delivery**																							
0	5,692	22.1	808	17.8	-15.4	14	5.4	-70.9	4978	30.7	27.7	139	11.2	-42.1	15	16.1	-21.7	19	21.1	-3.5	11	17.5	-16.6
1 - 10	10,968	42.7	1,521	33.5	-26.8	*		-151.1	9,327	57.6	42.7	272	21.9	-64.5	51	54.8	34.7	35	38.9	-10.9	*		-93.7
11 - 20	3,777	14.7	707	15.6	3.5	30	11.5	-13.2	1314	8.1	-29.4	249	20.0	20.0	22	23.7	32.4	15	16.7	7.7	*		-15.1
21 - 31	5,273	20.5	1,504	33.1	40.7	208	80.0	209.3	583	3.6	-76.1	584	47.0	82.4	*		-65.4	21	23.3	9.7	36	57.1	114.7
**Total opioid dispensings**																							
2	11,963	46.5	1,845	40.6	-16.8	49	18.9	-87.3	11,000	67.9	62.5	464	37.3	-26.6	52	55.9	26.7	43	47.8	3.5	21	33.3	-38.5
3	6,588	25.6	1,303	28.7	9.8	106	40.8	46.1	2,963	18.3	-25.1	383	30.8	16.3	19	20.4	-17.5	21	23.3	-7.5	22	34.9	28.8
4 - 5	4,290	16.7	783	17.3	2.1	54	20.8	14.8	1578	9.7	-29.2	232	18.7	7.3	17	18.3	5.9	20	22.2	19.8	*		-3.1
6+	2,869	11.2	609	13.4	9.7	51	19.6	33.4	661	4.1	-38.1	165	13.3	9.1	*		-29.8	*		-22.4	*		19.5

For privacy reasons, counts <11 are indicated by ""

© 2022 Esposito DB et al. *JAMA Network Open.*

Supplement 2: Characteristics of hydrocodone users by NOWS status

	Hydrocodone						
	All		No NOWS		NOWS		
	N	%	N	%	N	%	SD
**All**	**25,710**	**100.0**	**25,231**	**100.0**	**479**	**100.0**	
**Region**							
West	6,024	23.4	5926	23.5	98	20.5	-10.4
Central	8,384	32.6	8,233	32.6	151	31.5	-3.4
South	9,895	38.5	9,733	38.6	162	33.8	-14.0
Northeast	1,407	5.5	1,339	5.3	68	14.2	42.9
**Age category**							
<18	732	2.9	730	2.9	*		-27.5
18-24	11,103	43.2	10,969	43.5	134	28.0	-46.4
24-34	12,046	46.9	11,766	46.6	280	58.5	33.7
35+	1,829	7.1	1766	7.0	63	13.2	29.1
**Year of delivery**							
2000-2004	4,254	16.6	4219	16.7	35	7.3	-41.4
2005-2009	11,120	43.3	10,974	43.5	146	30.5	-38.5
2010-2014	10,336	40.2	10,038	39.8	298	62.2	65.1
**Race/ethnicity**							
White	18,725	72.8	18,317	72.6	408	85.2	44.1
Black	3,777	14.7	3751	14.9	26	5.4	-44.8
Hispanic/Latinx	1,448	5.6	1427	5.7	21	4.4	-8.3
Other	1,760	6.9	1736	6.9	24	5.0	-11.2
**Infant sex**							
Male	12,226	47.6	12,022	47.7	204	42.6	-14.4
Male/Female twins	567	2.2	552	2.2	15	3.1	8.3
Female	12,850	50.0	12,592	49.9	258	53.9	11.2
Other/unknown	67	0.3	65	0.3	*		3.9
**Multiparous**	19,707	76.7	19,320	76.6	387	80.8	14.6
**Multifetal gestation**	573	2.2	557	2.2	16	3.3	*9.7*
**Tobacco use**	2,585	10.1	2510	10.0	75	15.7	24.3
Current use	2,515	9.8	2442	9.7	73	15.2	*23.9*
Cessation treatment	157	0.6	153	0.6	*		*3.8*
History of use	129	0.5	124	0.5	*		*8.9*
**Maternal Morbidity Index**							
0	10,920	42.5	10,804	42.8	116	24.2	-56.8
1	6,591	25.6	6,491	25.7	100	20.9	-16.3
2	3,999	15.6	3884	15.4	115	24.0	30.8
3+	4,200	16.3	4,052	16.1	148	30.9	50.3
**Potential opioid indications**							
Abdominal pain	9,437	36.7	9,247	36.7	190	39.7	*8.8*
Arthritis arthropathies	5423	21.1	5259	20.8	164	34.2	42.9
Back and neck pain	8,444	32.8	8,188	32.5	256	53.4	61.4
Dental	2,460	9.6	2383	9.4	77	16.1	28.3
Infection	2,387	9.3	2346	9.3	41	8.6	*-3.7*
Joint pain	2,543	9.9	2456	9.7	87	18.2	34.7
Migraine headache	5,074	19.7	4974	19.7	100	20.9	*4.1*
Orthopedic injury	3,555	13.8	3463	13.7	92	19.2	21.0
Neuropathy/neuralgias	1699	6.6	1643	6.5	56	11.7	25.6
Autoimmune pain	112	0.4	108	0.4	*		*7.3*
Other chronic pain	1016	4.0	974	3.9	42	8.8	28.7
Other acute pain	3410	13.3	3305	13.1	105	21.9	33.1
**Psychiatric conditions**	5555	21.6	5398	21.4	157	32.8	36.5
ADHD	318	1.2	308	1.2	*		*9.7*
Anxiety	2,591	10.1	2512	10.0	79	16.5	27.4
Bipolar disorder	968	3.8	941	3.7	27	5.6	12.8
Depression	2,889	11.2	2805	11.1	84	17.5	26.0
Posttraumatic stress disorder	315	1.2	305	1.2	*		*9.8*

© 2022 Esposito DB et al. *JAMA Network Open.*

Schizophrenia	55	0.2	53	0.2	*		*5.3*
Sleep disorders	495	1.9	477	1.9	18	3.8	16.0
**Substance use**	645	2.5	628	2.5	21	4.4	14.7
Alcohol abuse dependence	239	0.9	230	0.9	*		*11.7*
Amphetamine use disorder	103	0.4	99	0.4	*		*8.1*
Cocaine use	98	0.4	96	0.4	*		*0.9*
Hallucinogen use	*		*		.	0.0	*-2.0*
Marijuana use	263	1.0	258	1.0	*		*0.3*
Sedative hypnotic use disorders	21	0.1	17	0.1	*		*16.2*
**Other comorbidities**							
Anemia	608	2.4	597	2.4	11	2.3	*-0.7*
Antiphospholipid syndrome	*		*		0	0.0	*-2.8*
Chronic pulmonary disease	2,651	10.3	2597	10.3	54	11.3	*4.5*
Diabetes	794	3.1	774	3.1	20	4.2	8.4
Gout	12	0.1	12	0.1	0	0.0	-4.5
Hypertension	1,240	4.8	1201	4.8	39	8.1	19.5
Infertility	44	0.2	42	0.2	*		6.5
Obesity overweight	1,016	4.0	993	3.9	23	4.8	6.0
Polycystic ovarian syndrome	58	0.2	58	0.2	0	0.0	-9.6
Renal disease	61	0.2	58	0.2	*		8.7
Thyroid disorders	744	2.9	730	2.9	14	2.9	0.3
**Medications during the 90 days before delivery**							
Atypical antipsychotics	36	0.1	35	0.1	*		2.4
Barbiturates	808	3.1	801	3.2	*		-16.1
Benzodiazepines, long acting	290	1.1	277	1.1	13	2.7	16.7
Benzodiazepines, short acting	1,634	6.4	1566	6.2	68	14.2	37.7
SNRI	234	0.9	575	2.3	27	5.6	24.5
SSRI	2,551	9.9	3272	13.0	77	16.1	12.5
Tricyclic antidepressants	231	0.9	219	0.9	15	3.1	22.9
Other antidepressants	602	2.3	2468	9.8	83	17.3	31.4
Other hypnotics	3,349	13.0	225	0.9	*		5.0
**Medications during the baseline period**							
Anti Nausea	11,162	43.4	10,933	43.3	229	47.8	12.7
Antibiotics	17,211	66.9	16,878	66.9	333	69.5	*8.0*
Anticonvulsants	1,692	6.6	1619	6.4	73	15.2	40.6
Antidepressants	6,062	23.6	5,874	23.3	188	39.3	49.5
Antidiabetic	358	1.4	354	1.4	*		*-7.5*
Antihypertensives	1,708	6.6	1657	6.6	51	10.7	20.6
Anxiolytics	460	1.8	444	1.8	16	3.3	14.2
Atypical APM	64	0.3	63	0.3	*		*-1.2*
Barbiturates	1,627	6.3	1603	6.4	24	5.0	-8.2
Benzodiazepines	3,727	14.5	3594	14.2	133	27.8	47.6
Corticosteroids	4,647	18.1	4555	18.1	92	19.2	4.2
Insulin	435	1.7	425	1.7	*		4.3
Mood Stabilizer	813	3.2	780	3.1	33	6.9	24.8
Other hypnotics	2,783	10.8	2708	10.7	75	15.7	20.7
Progestins	954	3.7	937	3.7	17	3.6	-1.2
Stimulants for ADHD	548	2.1	530	2.1	18	3.8	13.9
Suspected Teratogens	3,918	15.2	3845	15.2	73	15.2	0.0
Triptans	704	2.7	684	2.7	20	4.2	11.4
Typical APM	758	3.0	726	2.9	32	6.7	25.3
**High utilization**							
>120 MME for >90 days	16,843	65.5	16,439	65.2	404	84.3	64.0
>3 opioid prescribers	2,985	11.6	2875	11.4	110	23.0	43.9

16 *For privacy reasons, counts <11 are indicated by "*"

© 2022 Esposito DB et al. *JAMA Network Open.*

Supplement 3: Association Between Oxycodone and NOWS

	Oxycodone			*Hydrocodone*					
	Events	*Total*	*Risk*	*Events*	*Total*	*Risk*	**RR**	**LCL**	**UCL**
**Main analysis**									
Crude	331	4,540	0.073	479	25,710	0.019	3.91	3.41	4.49
PS weighted (opioid characteristics only)							2.46	2.17	2.79
PS weighted (all measured confounders)							1.90	1.69	2.15
PS weighted (all measured confounders + outcome model adjustment)[1]							1.87	1.66	2.11
**Sensitivity analyses**									
Days supply in the 90 days prior to delivery									
1 - 5[2]	<11	278	0.007	11	1,893	0.006	1.60	0.34	7.44
6 - 9	14	690	0.020	40	5,319	0.008	1.56	0.88	2.76
10 - 29	38	1,079	0.035	91	7,460	0.012	1.72	1.20	2.46
30 - 90[3]	277	2,493	0.111	337	11,038	0.031	1.82	1.58	2.10
Cumulative exposure in the 90 days prior to delivery									
First quartile (<390)	18	1,132	0.016	111	12,837	0.009	1.49	0.91	2.42
Second quartile (390-<900)	37	1,092	0.034	106	6,403	0.017	1.72	1.18	2.49
Third quartile (900-<2838.5)	95	1,181	0.080	189	5,341	0.035	1.83	1.45	2.32
Fourth quartile (≥2838.5)[4]	181	1,135	0.159	73	1,129	0.065	1.39	1.11	1.72
Timing of exposure									
≥30 days prior to delivery	14	660	0.021	55	4,797	0.011	0.92	0.53	1.59
<30 days prior to delivery	317	3,880	0.082	424	20,913	0.020	1.76	1.55	2.00
Alternative outcome definition									
Severe NOWS[1]	188	4,540	0.041	239	25,710	0.009	2.03	1.72	2.40
Alternative calculation of MME									
Applying a discount of 50% to MME conversion factor	331	4,540	0.073	479	25,710	0.019	1.99	1.82	2.18
Applying an increase of 50% to MME conversion factor	331	4,540	0.073	479	25,710	0.019	1.55	1.42	1.69
Alternative handling of the exposure propensity score									
1:1 greedy match in lieu of stratification and reweighting	218	3,904	0.056	128	3,904	0.033	1.70	1.38	2.10
Including all potential covariates without limiting to confounders	331	4,540	0.073	479	25,710	0.019	1.95	1.73	2.21

1: Outcome model also adjusted for long-acting benzodiazepine use and US region of residence
2: Outcome model also adjusted for antidepressants and anticonvulsant medications at baseline
3: Outcome model also adjusted for US region of residence
4: Outcome model also adjusted for US region of residence, long-acting benzodiazepine use, and race. Observed confounding could not be fully adjusted due to sample size limitations.
5: Outcome model also adjusted for US region of residence, long-acting benzodiazepine use, and race.

© 2022 Esposito DB et al. *JAMA Network Open.*

Supplement 4: Association Between Codeine and NOWS

	Codeine			*Hydrodocone*					
	Events	*Total*	*Risk*	*Events*	*Total*	*Risk*	**RR**	**LCL**	**UCL**
**Main analysis**									
Crude	88	16,202	0.005	479	25,710	0.019	0.29	0.23	0.37
PS weighted (opioid characteristics only)							0.54	0.42	0.68
PS weighted (all measured confounders)							0.57	0.46	0.70
**Sensitivity analyses**									
Days supply in the 90 days prior to delivery									
1 - 5[1]	18	3,278	0.005	17	3,009	0.006	1.61	0.79	3.31
6 - 9	34	8,781	0.004	52	8,626	0.006	0.65	0.42	1.00
10 - 29	31	7,468	0.004	120	13,216	0.009	0.46	0.31	0.68
30 - 90	35	6,479	0.005	656	20,331	0.032	0.44	0.31	0.62
Cumulative exposure in the 90 days prior to delivery									
First quartile (<157.5)	25	5,835	0.004	40	5,181	0.008	0.94	0.54	1.65
Second quartile (157.5-<225)	26	6,078	0.004	44	5,286	0.008	0.69	0.41	1.15
Third quartile (225-<355.5)	31	7,587	0.004	81	9,604	0.008	0.71	0.46	1.10
Fourth quartile (≥355.5)	36	6,512	0.006	710	25,114	0.028	0.40	0.29	0.57
Timing of exposure									
≥30 days prior to delivery	18	6,697	0.003	87	7,602	0.011	0.31	0.18	0.53
<30 days prior to delivery	100	19,315	0.005	788	37,583	0.021	0.63	0.50	0.78
Alternative outcome definition									
Severe NOWS	47	26,012	0.002	418	45,185	0.009	0.48	0.35	0.66
Alternative calculation of MME									
Applying a discount of 50% to MME conversion factor	88	16,202	0.005	479	25,710	0.019	0.30	0.25	0.36
Applying an increase of 50% to MME conversion factor	88	16,202	0.005	479	25,710	0.019	0.55	0.45	0.68
Alternative handling of the exposure propensity score									
1:1 greedy match in lieu of stratification and reweighting	105	20,062	0.005	179	20,062	0.009	0.59	0.46	0.74

1: Outcome model also adjusted for maternal morbidity index

© 2022 Esposito DB et al. *JAMA Network Open.*

Supplement 5: Association Between Tramadol and NOWS

	Tramadol			*Hydrodocone*					
	Events	*Total*	*Risk*	*Events*	*Total*	*Risk*	**RR**	**LCL**	**UCL**
**Main analysis**									
Crude	51	1,244	0.041	479	25,710	0.019	2.20	1.66	2.92
PS weighted (opioid characteristics only)							1.37	1.04	1.81
PS weighted (all measured confounders)							1.06	0.73	1.56
**Sensitivity analyses**									
Days supply in the 90 days prior to delivery									
1 - 5	0	<11	0.000	11	1,893	0.006	Not estimable		
6 - 9[1]	<11	56	0.071	40	5,319	0.008	5.95	2.21	16.04
10 - 29	<11	280	0.021	91	7,460	0.012	1.20	0.54	2.71
30 - 90	41	900	0.046	337	11,038	0.031	1.16	0.85	1.59
Cumulative exposure in the 90 days prior to delivery									
First quartile (<450)	<11	299	0.030	123	14,035	0.009	1.97	1.02	3.79
Second quartile (450-<900)	<11	311	0.023	94	5,205	0.018	0.74	0.35	1.56
Third quartile (900-<1565)	<11	323	0.019	101	3,008	0.034	0.60	0.27	1.37
Fourth quartile (≥1565)	29	311	0.093	161	3,462	0.047	2.14	1.46	3.14
Timing of exposure									
≥30 days prior to delivery	<11	90	0.011	55	4,797	0.011	0.54	0.08	3.87
<30 days prior to delivery	50	1,154	0.043	424	20,913	0.020	1.25	0.95	1.66
Alternative outcome definition									
Severe NOWS	30	1,244	0.024	239	25,710	0.009	1.34	0.93	1.93
Alternative calculation of MME									
Applying a discount of 50% to MME conversion factor	51	1,244	0.041	479	25,710	0.019	1.20	0.91	1.59
Applying an increase of 50% to MME conversion factor[6]	51	1,244	0.041	479	25,710	0.019	1.08	0.86	1.36
Alternative handling of the exposure propensity score									
1:1 greedy match in lieu of stratification and reweighting	74	2,074	0.036	69	2,074	0.033	1.07	0.78	1.48
Including all potential covariates without limiting to confounders	51	1,244	0.041	479	25,710	0.019	1.19	0.90	1.57

1: Outcome model also adjusted for anxiolytic and stimulant medication use. Adjustment for SNRI use was infeasible.

© 2022 Esposito DB et al. *JAMA Network Open.*

Supplement 6: Association Between Methadone and NOWS

	Methadone			*Hydrodocone*					
	Events	*Total*	*Risk*	*Events*	*Total*	*Risk*	**RR**	**LCL**	**UCL**
**Main analysis**									
Crude	94	260	0.362	479	25,710	0.019	19.41	16.14	23.33
PS weighted (opioid characteristics only)							3.53	2.87	4.34
PS weighted (all measured confounders)							3.22	2.56	4.05
PS weighted (all measured confounders + outcome model adjustment)[1]							3.02	2.45	3.73
**Sensitivity analyses**									
Days supply in the 90 days prior to delivery									
1 - 5	<11	<11	1.000	11	1,893	0.006		Not estimable	
6 - 9	<11	<11	1.000	40	5,319	0.008		Not estimable	
10 - 29	<11	11	0.273	91	7,460	0.012		Not estimable	
30 - 90[2]	88	246	0.358	337	11,038	0.031	2.86	2.27	3.61
Cumulative exposure in the 90 days prior to delivery									
First quartile (<4245)[3]	18	65	0.277	458	25,438	0.018	4.82	3.25	7.15
Second quartile (4245-<7545)[4]	22	65	0.338	18	247	0.073	3.36	1.48	7.64
Third quartile (7545-<12267)	20	65	0.308	<11	23	0.130		Not estimable	
Fourth quartile (≥12267)	34	65	0.523	0	2	0.000		Not estimable	
Timing of exposure									
≥30 days prior to delivery	<11	<11	0.800	55	4,797	0.011		Not estimable	
<30 days prior to delivery[5]	90	255	0.353	424	20,913	0.020	2.17	1.85	2.53
Alternative outcome definition									
Severe NOWS[7]	44	260	0.169	239	25,710	0.009	4.06	2.68	6.16
Alternative calculation of MME									
Applying a discount of 50% to MME conversion factor	94	260	0.362	479	25,710	0.019	3.96	3.24	4.83
Applying an increase of 50% to MME conversion factor	94	260	0.362	479	25,710	0.019	2.63	2.16	3.21
Alternative handling of the exposure propensity score									
1:1 greedy match in lieu of stratification and reweighting							8.00	2.91	21.97
Including all potential covariates without limiting to confounders	94	260	0.362	479	25,710	0.019	3.89	3.09	4.89

1: Outcome model also adjusted for potential doctor/pharmacy shopping. Additional adjustment for SNRI use was infeasible.
2: Outcome model also adjusted for substance use disorders, tobacco use, sleep disorders, and barbiturate use. Additional adjustment was infeasible.
3: Outcome model also adjusted for use of mood stabilizers and depression
4: Outcome model also adjusted for use of short acting benzodiazepines and depression. Additional adjustment was infeasible.
5: Outcome model also adjusted for use of hypnotics and depression. Additional adjustment was infeasible.
6: Outcome model also adjusted for race, sleep disorders, and use of barbiturates. Additional adjustment was infeasible.
7: Outcome model also adjusted for long-acting benzodiazepines and year

© 2022 Esposito DB et al. *JAMA Network Open.*

Supplement 7: Association Between Morphine and NOWS

	Morphine			*Hydrodocone*					
	Events	*Total*	*Risk*	*Events*	*Total*	*Risk*	**RR**	**LCL**	**UCL**
**Main analysis**									
Crude	15	63	0.238	479	25,710	0.019	12.78	8.14	20.05
PS weighted (opioid characteristics only)							5.85	3.62	9.46
PS weighted (all measured confounders)							2.79	1.22	6.41
PS weighted (all measured confounders + outcome model adjustment)[1]							2.84	1.30	6.22
**Sensitivity analyses**									
Days supply in the 90 days prior to delivery									
1 - 5	0	<11	0.000	11	1,893	0.006	Not estimable		
6 - 9	0	<11	0.000	40	5,319	0.008	Not estimable		
10 - 29	0	<11	0.000	91	7,460	0.012	Not estimable		
30 - 90[2]	15	50	0.300	337	11,038	0.031	2.55	1.33	4.89
Cumulative exposure in the 90 days prior to delivery									
First quartile (<2152)	0	16	0.000	363	23,645	0.015	Not estimable		
Second quartile (2152-<4186)	<11	16	0.125	95	1,782	0.053	Not estimable		
Third quartile (4186-<10650)	<11	16	0.375	21	278	0.076	Not estimable		
Fourth quartile (≥10650)	<11	15	0.467	0	5	0.000	Not estimable		
Timing of exposure									
≥30 days prior to delivery	0	<11	0.000	55	4,797	0.011	Not estimable		
<30 days prior to delivery[3]	15	57	0.263	424	20,913	0.020	2.12	1.03	4.38
Alternative outcome definition									
Severe NOWS[4]	<11	63	0.127	239	25,710	0.009	3.00	1.33	6.80
Alternative calculation of MME									
Applying a discount of 50% to MME conversion factor	15	63	0.238	479	25,710	0.019			
Applying an increase of 50% to MME conversion factor	15	63	0.238	479	25,710	0.019	1.33	0.78	2.37
Alternative handling of the exposure propensity score									
1:1 greedy match in lieu of stratification and reweighting							2.33	0.70	7.82
Including all potential covariates without limiting to confounders	15	63	0.238	479	25,710	0.019	1.63	0.83	3.23

1: Outcome model also adjusted for SNRI, long and short acting benzodiazepine, mood stabilizer and hypnotic use, sleep disorders, and potential doctor/pharmacy shopping.
2: Outcome model also adjusted for mood stabilizer, antibiotic, anxiolytic, hypnotic, and antidiabetic medication use and hypertension. Additional adjustment was infeasible.
3: Outcome model also adjusted for comorbidity score. Additional adjustment was infeasible.
4: Outcome model also adjusted for benzodiazepine and mood stabilizer use, sleep disorders, and possible doctor/pharmacy shopping. Additional adjustment was infeasible.

© 2022 Esposito DB et al. *JAMA Network Open.*

Supplement 8: Association Between Agonist Strength and NOWS

	Full strong agonist			*Full weak agonist*					
	Events	*Total*	*Risk*	*Events*	*Total*	*Risk*	**RR**	**LCL**	**UCL**
**Main analysis**									
Crude	451	5,046	0.089	618	43,156	0.014	6.24	5.55	7.02
PS weighted (opioid characteristics only)							2.70	2.43	2.99
PS weighted (all measured confounders)							1.97	1.78	2.17
PS weighted (all measured confounders + outcome model adjustment)[1]							1.92	1.73	2.12
**Sensitivity analyses**									
Days supply in the 90 days prior to delivery									
<10	<11	296	0.014	23	4,061	0.006	2.86	0.98	8.36
10 - 29	16	732	0.022	66	11,179	0.006	1.79	1.07	2.98
30 - 90[3]	43	1,162	0.037	122	12,186	0.010	1.99	1.43	2.78
Cumulative exposure in the 90 days prior to delivery									
First quartile (<412.5)									
Second quartile (412.5-<975)	12	446	0.027	83	12,793	0.006	2.64	1.47	4.73
Third quartile (975-<3375)	29	1,151	0.025	112	10,677	0.010	1.85	1.25	2.74
Fourth quartile (<3375)	408	3,196	0.128	374	11,229	0.033	1.92	1.70	2.18
Timing of exposure									
≥30 days prior to delivery	20	698	0.029	71	9,453	0.008	1.70	1.08	2.70
<30 days prior to delivery	431	4,348	0.099	547	33,703	0.016	2.02	1.81	2.25
Alternative outcome definition									
Severe NOWS[1]	249	5,046	0.049	310	43,156	0.007	2.69	2.33	3.09
Alternative calculation of MME									
Applying a discount of 50% to MME conversion factor	451	5,046	0.089	618	43,156	0.014	1.92	1.73	2.12
Applying an increase of 50% to MME conversion factor	451	5,046	0.089	618	43,156	0.014	2.86	0.98	8.36
Alternative handling of the exposure propensity score									
1:1 greedy match in lieu of stratification and reweighting	237	4,203	0.056	126	4,203	0.030	1.88	1.53	2.31

1: Outcome model also adjusted for long-acting benzodiazepine use

© 2022 Esposito DB et al. *JAMA Network Open.*

Supplement 9: Association Between Opioid Half-Life (Medium vs. Short) and NOWS

	Medium half life			*Short half life*					
	Events	*Total*	*Risk*	*Events*	*Total*	*Risk*	RR	LCL	UCL
**Main analysis**									
Crude	820	30,340	0.027	103	16,265	0.006	4.27	3.48	5.23
PS weighted (opioid characteristics only)							1.03	0.92	1.15
PS weighted (all measured confounders)							0.95	0.85	1.06
PS weighted (all measured confounders + outcome model adjustment)							0.95	0.85	1.06
**Sensitivity analyses**									
Days supply in the 90 days prior to delivery									
<10	78	9,553	0.008	41	9,130	0.004	1.06	0.76	1.46
10 - 29	131	8,568	0.015	25	4,452	0.006	1.70	1.19	2.44
30 - 90[1]	611	12,219	0.050	37	2,683	0.014	0.94	0.78	1.12
Cumulative exposure in the 90 days prior to delivery									
First quartile (<240)	56	7,589	0.007	41	8,932	0.005	0.89	0.63	1.27
Second quartile (240-<437.5)	90	7,580	0.012	20	5,069	0.004	2.51	1.58	3.99
Third quartile (437.5-<1050)	166	7,504	0.022	20	1,846	0.011	1.58	1.04	2.38
Fourth quartile (≥1050)[2]	508	7,667	0.066	22	418	0.053	0.92	0.64	1.32
Timing of exposure									
≥30 days prior to delivery	71	5,472	0.013	15	4,572	0.003	1.42	0.97	2.07
<30 days prior to delivery	749	24,868	0.030	88	11,693	0.008	0.86	0.77	0.97
Alternative outcome definition									
Severe NOWS	435	30,340	0.014	49	16,265	0.003	0.93	0.79	1.08
Alternative calculation of MME									
Applying a discount of 50% to MME conversion factor	820	30,340	0.027	103	16,265	0.006	1.10	0.99	1.22
Applying an increase of 50% to MME conversion factor	820	30,340	0.027	103	16,265	0.006	1.03	0.92	1.14
Alternative handling of the exposure propensity score									
1:1 greedy match in lieu of stratification and reweighting	133	12,969	0.010	91	12,969	0.007	1.46	1.12	1.90

1: Outcome model also adjusted for baseline triptan use, region, and total opioid dispensings
2: Outcome model also adjusted for age, infant gender, exposure within 30 days of delivery, and extended use of high doses

© 2022 Esposito DB et al. *JAMA Network Open.*

Supplement 10: Association Between Opioid Half-Life (Long vs. Short) and NOWS

	Long half life			*Short half life*					
	Events	***Total***	***Risk***	***Events***	***Total***	***Risk***	**RR**	**LCL**	**UCL**
**Main analysis**									
Crude	146	1,597	0.091	103	16,265	0.006	14.44	11.28	18.48
PS weighted (opioid characteristics only)							1.38	1.17	1.62
PS weighted (all measured confounders)							1.33	1.12	1.56
PS weighted (all measured confounders + outcome model adjustment)[1]							1.33	1.13	1.57
**Sensitivity analyses**									
Days supply in the 90 days prior to delivery									
<10	<11	101	0.069	41	9,130	0.004	6.72	2.99	15.09
10 - 29	<11	328	0.027	25	4,452	0.006	3.29	1.59	6.78
30 - 90[2]	130	1,168	0.111	37	2,683	0.014	1.22	0.99	1.48
Cumulative exposure in the 90 days prior to delivery									
First quartile (<450)	13	364	0.036	61	14,041	0.004	3.89	2.22	6.82
Second quartile (450-<1040)[3]	<11	434	0.021	20	1,805	0.011	2.88	1.11	7.50
Third quartile (1040-<2160)[4]	24	398	0.060	<11	295	0.014	2.52	0.96	6.63
Fourth quartile (≥2160)[5]	100	401	0.249	18	124	0.145	0.80	0.57	1.12
Timing of exposure									
≥30 days prior to delivery[6]	<11	107	0.047	15	4,572	0.003	4.05	1.26	13.04
<30 days prior to delivery[7]	141	1,490	0.095	88	11,693	0.008	1.35	1.14	1.60
Alternative outcome definition									
Severe NOWS[8]	75	1,597	0.047	49	16,265	0.003	1.14	0.90	1.44
Alternative calculation of MME									
Applying a discount of 50% to MME conversion factor	166	1,923	0.086	114	19,177	0.006	1.25	1.07	1.47
Applying an increase of 50% to MME conversion factor	166	1,923	0.086	114	19,177	0.006	1.64	1.40	1.92
Alternative handling of the exposure propensity score									
1:1 greedy match in lieu of stratification and reweighting	54	1,022	0.053	30	1,022	0.029	1.80	1.17	2.77

1: Outcome model also adjusted for baseline antidepressant and triptan use
2: Outcome model also adjusted for baseline triptan use and exposure 60-90 days before delivery
3: Outcome model also adjusted for total opioid dispensings and short acting benzodiazepine use
4: Outcome model also adjusted for antidepressant and benzodiazepine use, comorbidity score, and >3 opioid prescribers
5: Outcome model also adjusted for
6: Outcome model also adjusted for mood stabalizer use
7: Outcome model also adjusted for triptan and anxiolytic medication use
8: Outcome model also adjusted for baseline triptan and antidepressant use

© 2022 Esposito DB et al. *JAMA Network Open.*
